# JrCDPK13L-mediated phosphorylation of JrERF113L promotes walnut resistance to *Colletotrichum gloeosporioides*

**DOI:** 10.1093/plphys/kiag494

**Published:** 2026-07-13

**Authors:** Haiyi Yu, Yuhui Dong, Kun Zhang, Jing Zhao, Xinyu Bing, Yuheng Liu, Fei Yang, Weichen Song, Hongyu Zhu, Di Wu, Jianning Liu, Qiang Liang, Ke Qiang Yang, Hongcheng Fang

**Affiliations:** College of Forestry, Shandong Agricultural University, Tai’an, Shandong 271000, China; College of Forestry, Shandong Agricultural University, Tai’an, Shandong 271000, China; College of Forestry, Shandong Agricultural University, Tai’an, Shandong 271000, China; College of Forestry, Shandong Agricultural University, Tai’an, Shandong 271000, China; College of Forestry, Shandong Agricultural University, Tai’an, Shandong 271000, China; College of Forestry, Shandong Agricultural University, Tai’an, Shandong 271000, China; College of Forestry, Shandong Agricultural University, Tai’an, Shandong 271000, China; College of Forestry, Shandong Agricultural University, Tai’an, Shandong 271000, China; College of Forestry, Shandong Agricultural University, Tai’an, Shandong 271000, China; College of Forestry, Shandong Agricultural University, Tai’an, Shandong 271000, China; College of Forestry, Shandong Agricultural University, Tai’an, Shandong 271000, China; College of Forestry, Shandong Agricultural University, Tai’an, Shandong 271000, China; College of Forestry, Shandong Agricultural University, Tai’an, Shandong 271000, China; College of Forestry, Shandong Agricultural University, Tai’an, Shandong 271000, China

## Abstract

Walnut anthracnose caused by *Colletotrichum gloeosporioides* poses a serious threat to the walnut industry. Calcium-dependent protein kinases (CDPKs) are critical regulators that transmit calcium (Ca^2+^) signals into cellular immune responses. However, the specific functional mechanisms of CDPKs in walnut resistance to anthracnose infection remain unclear. In this study, we identified and characterized JrCDPK13L as a positive regulator of anthracnose resistance in walnut, acting in a Ca^2+^-dependent manner through transcriptomic, genetic, and molecular biology assays. Mechanistically, JrCDPK13L interacts with the ethylene-responsive factor JrERF113L, which was shown to promote walnut resistance to anthracnose based on disease resistance evaluation in transgenic strains. Furthermore, JrCDPK13L phosphorylates JrERF113L at Ser271/280 residues, which enhances both the protein stability and disease resistance of JrERF113L. In addition, JrERF113L directly binds to the promoter regions and activates the transcription of pathogenesis-related gene *JrPR5L*, and this activation is further strengthened by the JrCDPK13L-mediated phosphorylation of JrERF113L at Ser271/280. Collectively, our findings reveal a novel signaling module, JrCDPK13L-JrERF113L-JrPR5L, that enhances walnut resistance to anthracnose, providing significant insights into the role of Ca^2+^ signaling pathways in woody plant immunity.

## Introduction

Plant innate immune system consists of two principal layers: pattern-triggered immunity (PTI) and effector-triggered immunity (ETI). PTI is activated by cell surface pattern-recognition receptors (PRRs) upon detection of pathogen-associated molecular patterns (PAMPs), whereas ETI is triggered by intracellular nucleotide-binding domain leucine-rich repeat-containing receptors (NLRs) upon recognition of specific pathogen effectors (Jones and Dangl 2006; Dodds and Rathjen 2010). These two immune branches are interconnected and complementary, converging on downstream responses including Ca^2+^ influx, reactive oxygen species (ROS) burst, and MAPK activation, ultimately leading to systemic acquired resistance (SAR) (Ngou et al. 2021; Yuan et al. 2021). Ca^2+^ serves as a common downstream signal in both PTI and ETI pathways and is integral to their synergistic interaction (Huang et al. 2023). Plant immunity relies on dynamic changes in intracellular Ca^2+^ levels and the subsequent binding of Ca^2+^ to sensor proteins, including calmodulin proteins (CaMs), calcineurin B-like proteins (CBLs), CBL-interacting protein kinases (CIPKs), and calcium-dependent protein kinases (CDPKs or CPKs), which transmit Ca^2+^ signals and induce downstream defense responses (Yip Delormel and Boudsocq 2019; Tang et al. 2020; Zhang et al. 2024; Wang 2024a  Wang et al. 2024b).

Among these calcium sensors, CDPKs/CPKs are distinctive as they integrate Ca^2+^ sensing and downstream response activities within a single molecule (Hu et al. 2024). A typical CDPK contains an N-terminal variable domain, a serine/threonine kinase domain, an auto-inhibitory junction domain, and a C-terminal calmodulin-like domain (CaM-LD) harboring EF-hand motifs for Ca^2+^ binding. An increase in Ca^2+^ concentration triggers a conformational change in CDPK, relieving auto-inhibition and activating its kinase activity (Liese et al. 2024). Previous studies have revealed that the phosphorylation of CDPK28 at Ser318 induces conformational changes, enabling the kinase to remain active at low Ca^2+^ concentrations and thus allowing rapid immune responses (Bredow et al. 2021). Furthermore, CDPKs are pivotal regulators of plant immune that translate calcium signals into cellular responses by phosphorylating diverse substrate proteins (Fan et al. 2023). For instance, OsCPK17 interacts with and phosphorylates OsRLCK176 at *Ser83*, which promotes OsRLCK176 accumulation and enhances rice resistance to *Xanthomonas oryzae* pv. *Oryzae* (Mou et al. 2024). Recent research indicates that heat stress induces OsCDPK24 and OsCDPK28 to phosphorylate serine 146 of OsHSFA4d, which improves thermo tolerance and upregulates cellulose synthase-like F6 (*CslF6*) expression, thereby reducing disease resistance (Fang et al. 2025). Thus, in-depth investigation of the phosphorylation mechanisms of CDPKs and their substrate proteins is of great importance for elucidating the molecular basis of plant disease resistance.

The AP2/ERF (APETALA2/ethylene-responsive factor) transcription factors (TFs) contain one or two AP2/ERF DNA-binding domains and typically bind to GCC-box (AGCCGCC) or DRE/CRT (A/GCCGAC) cis-elements in promoter regions to regulate genes involved in plant immunity (Licausi et al. 2013; Feng et al. 2020). For example, MdERF61 directly binds to GCC-boxes in the mdm-miR397b promoter and represses mdm-miR397b transcription, which impacts apple resistance to *Fusarium solani* by regulating the *mdm-miR397b-MdLAC7b* module (Zhou et al. 2024). Upon infection by *Magnaporthe oryzae* or *Xanthomonas oryzae* pv. *Oryzae*, the binding of OsEIL3 to the *OsERF040* promoter is enhanced, whereas its binding to the *OsWRKY28* promoter is weakened, which promotes the biosynthesis and signaling of salicylic acid and jasmonic acid, and boosts disease resistance in rice (Zhu et al. 2024). In addition, the role of post-translational modifications of ERF TFs in plant immunity has been widely reported. MPK3/MPK6 phosphorylates AtERF72 at Serine 151 and enhances its transcriptional activity, thereby activating defense genes and increasing plant resistance to *Botrytis cinerea* (Li et al. 2022). Similarly, the GmMKK4-GmMPK6 cascade phosphorylates GmERF113, stabilizing the protein and elevating the expression of pathogenesis-related (*PR*) genes to confer resistance to *Phytophthora sojae* in soybean (Gao et al. 2022). Therefore, it is necessary to make a profound study on the role of ERF TFs in plant immunity, both at the transcriptional and protein levels.

Walnut anthracnose caused by *Colletotrichum gloeosporioides* has become a devastating disease affecting the walnut industry, causing yield losses of 30 to 50% in severe cases and seriously restricting the healthy development of this industry (Zhu et al. 2015; Yang et al. 2023). In recent years, the rapid development of *Juglans* genomics research, coupled with the deep integration of genetic transformation, gene editing and molecular biology (Yan et al. 2021; Song et al. 2025), has provided valuable bioinformatics resources and technology platforms for further elucidating the regulatory mechanisms of walnut resistance to anthracnose. Dual transcriptome analysis between walnut and *C. gloeosporioides* revealed that *C. gloeosporioides* secretes effector proteins in a stage-specific manner, while the walnut host exhibits genotype-specific defense responses, with resistant lines displaying a more comprehensive response (Li et al. 2024). Using transcriptomics and functional genomics, our previous work identified JrWRKY21-JrPTI5L-JrPR5L, lncRNA109897-JrCCR4-JrTLP1b, and JrPHL8-JrWRKY4-JrSTH2L as mediators of walnut resistance to *C. gloeosporioides* (Zhou et al. 2022, 2023; Mu et al. 2024). Additionally, genome-wide association studies (GWAS) of walnut resistance to anthracnose have identified significant SNP markers associated with anthracnose resistance and revealed the defense functions of *JrWDRC2A9* and *JrGPIAP* (Gong et al. 2025). However, a full understanding of the precise mechanisms by which Ca^2+^ signals are transduced during walnut resistance to anthracnose remains lacking.

In this study, we demonstrate that *C. gloeosporioides* induces Ca^2+^ accumulation and the upregulates the expression of *JrCDPK13L*, which enhances walnut resistance to anthracnose. JrCDPK13L interacts with JrERF113L in vitro and in vivo and phosphorylates JrERF113L at Ser271/280, thereby improving the protein stability of JrERF113L. Furthermore, JrERF113L binds to the GCCGAC motif in the *JrPR5L* promoter and promotes its transcription. Notably, JrCDPK13L-mediated phosphorylation of JrERF113L at Ser271/280 facilitates the transcriptional activity of *JrPR5L* and walnut resistance to anthracnose. Collectively, our findings elucidate a novel molecular mechanism by which JrCDPK13L enhances walnut resistance to anthracnose through phosphorylating and activating JrERF113L-JrPR5L pathway.

## Results

### JrCDPK13L responds to *C. gloeosporioides* infection through Ca^2+^ signaling

**Figure 1:**
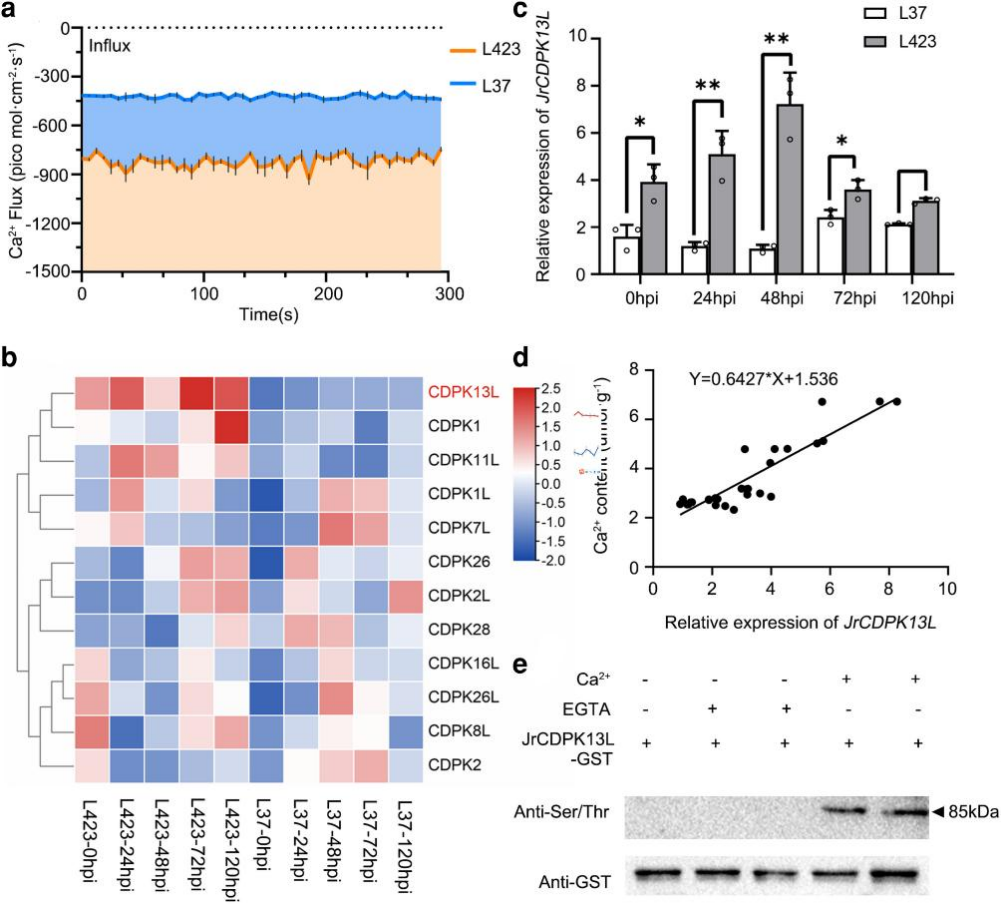
JrCDPK13Lresponds to *C. gloeosporioides* infection through Ca^2+^ signaling. a) Intracellular free Ca^2+^ dynamics in the resistant (L423) and susceptible (L37) walnut leaves at 48 h post-inoculation(hpi) with *Colletotrichum gloeosporioides*. b) Heatmap showing the differential expression profiles of the calcium-dependent protein kinase (CDPK) gene family in L423 vs. L37 at different time points after inoculation with *C. gloeosporioides*, as determined by RNA-seq. c) Relative expression levels of *JrCDPK13L* in L423 vs. L37 walnut leaves at different time points after inoculation with *C. gloeosporioides*, as detected by qRT-PCR using 18S rRNA as the internal reference gene. d) Correlation analysis between Ca^2+^ content and the relative expression of *JrCDPK13L* in walnut leaves during infection with *C. gloeosporioides*. e) Autophosphorylation activity of JrCDPK13L depend on Ca^2+^ was analyzed using Ser/Thr antibody. Data are presented as means ± SD of three biological replicates. Asterisks indicate significant differences compared with the control (two-tailed Student's *t*-test, *P < 0.05, **P < 0.01).

Our previous transcriptome analysis on the infection of *C. gloeosporioides* on walnut leaves showed that the enzyme activity and hormone content in resistant leaves (L423) were higher than those in susceptible leaves (L37) (Zhou et al. 2022). In this study, we also determined that the intracellular free Ca^2+^ dynamic in L423 was significantly higher than that in L37 during the infection process of *C. gloeosporioides* using noninvasive micro-test technology (NMT) (Fig. 1a). CDPKs/CPKs are key regulators of plant immunity that translate calcium signals into cellular responses (Fan et al. 2023). Therefore, the differential expression analysis during the infection process of *C. gloeosporioides* identified 12 CDPK genes, among which JrCDPK13L (LOC108994928) was the most abundantly expressed CDPK gene, with its expression significantly upregulated in L423 (Fig. 1b, Table S1). Further quantitative analysis using qRT-PCR confirmed a significant increase in *JrCDPK13L* transcript levels in resistant leaves compared with the susceptible leaves (Fig. 1c). Subsequently, we focused on JrCDPK13L for further research. Protein amino acid sequence alignment indicated that JrCDPK13L has the highest identity with MaCDPK in azedarach (*Melia azedarach*), and FcCDPK13L in Japanese beech (*Ficus carica*) (Figure S1a, b). Further sequence analysis showed that JrCDPK13L has three intact EF-hands (EF3) and one degenerated (Figure S1c). In this study, we first found a positive correlation between the transcription level of *JrCDPK13L* and the dynamic changes in Ca^2+^ content (Fig. 1d). In addition, to explore whether the calcium affects kinase activity of JrCDPK13L, we conducted the kinase assay using Ser/Thr antibody, and found that JrCDPK13L had autophosphorylation activity after adding free Ca^2+^, but the kinase activity disappears after adding EGTA (Fig. 1e). These results indicated that JrCDPK13L relied on Ca^2+^ signaling to respond to the infection of *C. gloeosporioides.*

### JrCDPK13L enhances walnut resistance to *C. gloeosporioides*

**Figure 2:**
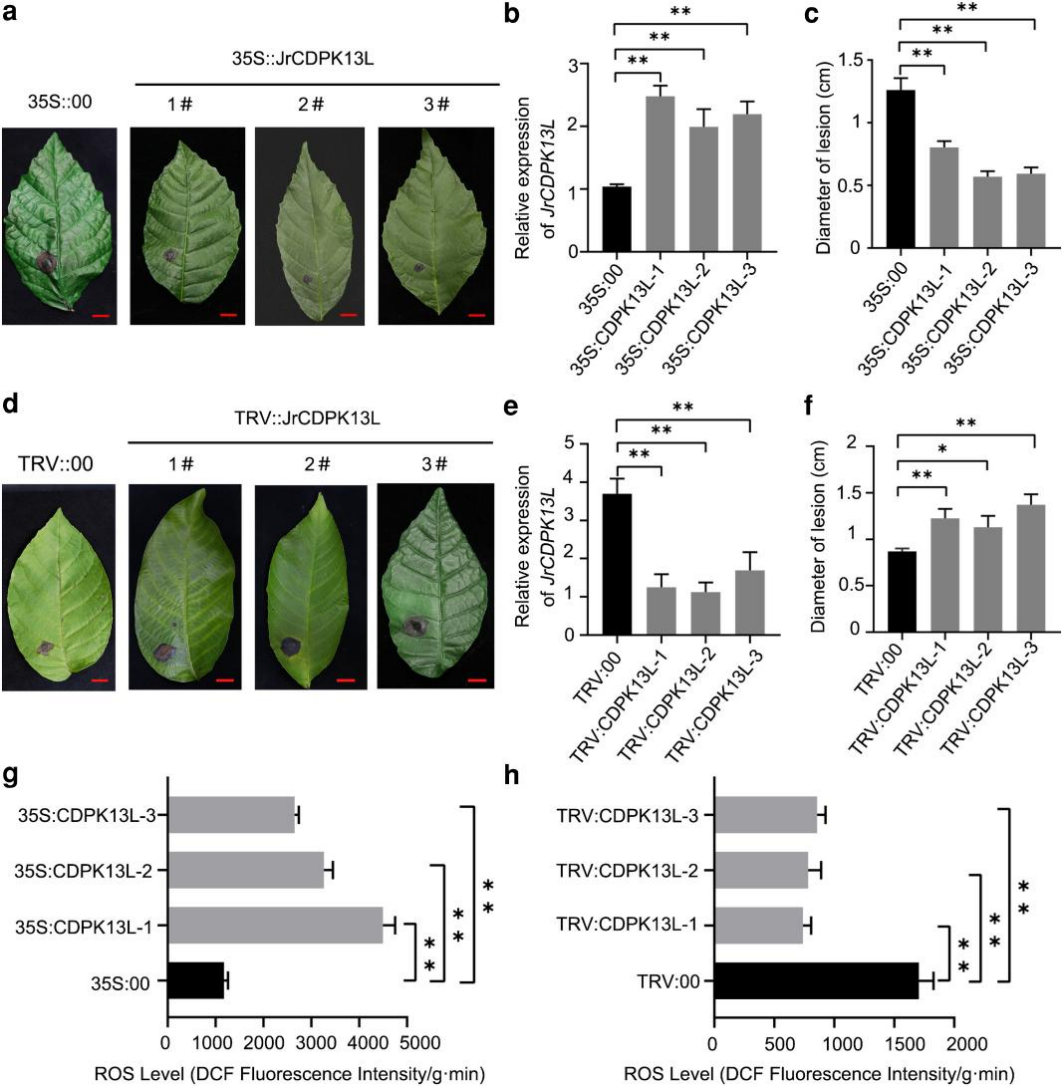
JrCDPK13L enhances walnut resistance to *C. gloeosporioides*. a) Leaf phenotypes of *35S::JrCDPK13L* and *35S::00* walnut leaves infected with *C. gloeosporioides*. Bars = 1 cm. b) The relative expression levels of *JrCDPK13L* in *35S::JrCDPK13L* and *35S::00* walnut leaves. 18S rRNA was amplified as an internal control. c) Diameter of lesions in *35S::JrCDPK13L* and *35S::00* walnut leaves. d) Leaf phenotypes of *TRV::JrCDPK13L* and *TRV::00* walnut leaves infected with *C. gloeosporioides*. Bars = 1 cm. e) The relative expression levels of *JrCDPK13L* in *TRV::JrCDPK13L* and *TRV::00* walnut leaves. 18S rRNA was amplified as an internal control. f) Diameter of lesions in *TRV::JrCDPK13L* and *TRV::00* walnut leaves. g) ROS levels in *35S::JrCDPK13L* and *35S::00* walnut leaves. h) ROS levels in *TRV::JrCDPK13L* and *TRV::00* walnut leaves. Data are presented as means ± SD of three biological replicates. Asterisks indicate significant differences compared with the control (two-tailed Student's *t*-test, *P < 0.05, **P < 0.01).

To investigate the function of JrCDPK13L in walnut resistance to anthracnose, we ligated the full-length coding sequence (CDS) of *JrCDPK13L* into the Pzp211 empty vector (negative control) to generate the JrCDPK13L-pZP211 recombinant plasmid. The recombinant plasmids were transformed into “B37” walnut leaves via vacuum infiltration, resulting in transient overexpression of *JrCDPK13L* (*35S::JrCDPK13L*) (Fig. 2a). *JrCDPK13L* was successfully transferred to walnut leaves and checked by both PCR (Figure S2a) and immunoblotting (Figure S2b). Meanwhile, qRT-PCR analysis confirmed significantly higher *JrCDPK13L* expression levels in *35S::JrCDPK13L* transgenic plants than in controls (Fig. 2b). Subsequently, detached leaves from both *35S::00* (empty vector control) and *35S::JrCDPK13L* plants were inoculated with *C. gloeosporioides* strains “m9” using in vitro leaf inoculation assay. The *35S::JrCDPK13L* leaves exhibited significantly milder disease symptoms (Fig. 2a) and smaller lesion diameters (Fig. 2c) compared to the control leaves. Concurrently, we designed a JrCDPK13L-specific fragment for VIGS-mediated silencing in walnut plants (*TRV::JrCDPK13L*). Using primers pTRV1-F/R and pTRV2-F/JrCDPK13L-R, we then detected the expected 327-bp band in the TRV::JrCDPK13L samples. In contrast, this band was not amplified in TRV::00 (empty TRV vector control) samples using primers pTRV2-F/JrCDPK13L-R (Figure S3). qRT-PCR analysis further revealed that *JrCDPK13L* expression in *TRV::JrCDPK13L* walnut leaves was significantly lower than in the control group, confirming the successful insertion of the JrCDPK13L-specific fragment and effective gene silencing (Fig. 2e). Compared to the control (*TRV::00*), *TRV::JrCDPK13L* transgenic walnut leaves displayed aggravated disease symptoms (Fig. 2d) and larger lesion diameters (Fig. 2f) following pathogen inoculation.

To further clarify whether JrCDPK13L functions in plant immunity, ROS generation and *PRs* gene expression were detected in the wild-type and *JrCDPK13L* transgenic lines. Compared with *35S::00* leaves, the ROS level in *35S::JrCDPK13L* leaves was significantly increased (Fig. 2g). On the contrary, the ROS production in *TRV::JrCDPK13L* is significantly lower than that in *TRV::00* (Fig. 2h). Previous studies have shown that *JrPR1*, *JrPR1L*, *JrPR5*, *JrPR5L*, and *JrSTH2L* served as the marker immune genes for walnut resistance to anthracnose (Zhou et al. 2022; Mu et al. 2024). As envisioned, *PR*s gene expression in *35S::JrCDPK13L* leaves were significantly higher than that in *35S::00* leaves, whereas the opposite was observed in *TRV::JrCDPK13L* vs. *TRV::00* (Figure S4). These results demonstrated that *JrCDPK13L* positively regulates walnut resistance to *C. gloeosporioides*.

### JrCDPK13L interacts with JrERF113L

**Figure 3:**
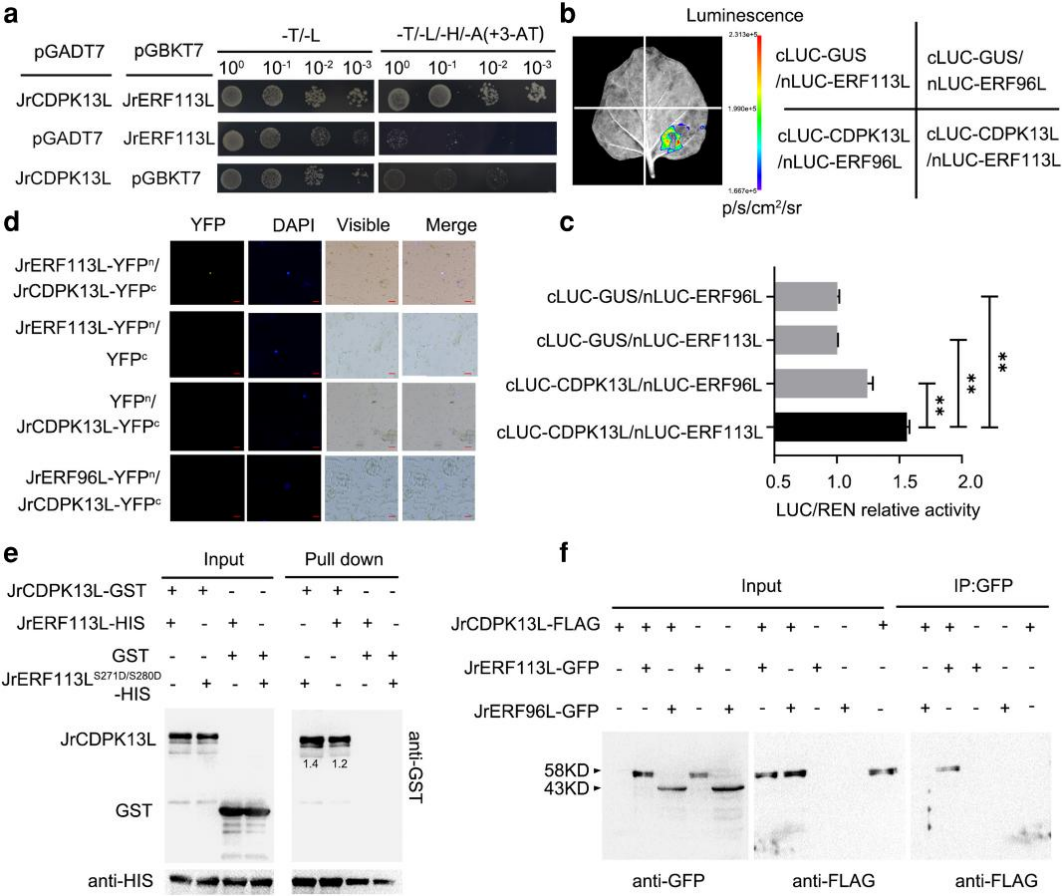
JrCDPK13L interacts with JrERF113L. a) Yeast two-hybrid (Y2H) assay indicates that JrCDPK13L interacts with JrERF113L. b) The luciferase complementation imaging assays detected the interaction between JrCDPK13L and JrERF113L in vivo. The pseudocolor scale bar represents the range of the luminescence intensity. c) LUC/REN relative activity of tobacco leaves infected by different vectors. () Bimolecular fluorescence complementation (BiFC) assays confirm the interaction between JrCDPK13L and JrERF113L in the nucleus of *N. benthamiana* epidermal cells. Scale bar = 2 μm. e) Pull-down assay shows the direct interaction of JrCDPK13L with JrERF113L in vitro. Immunoblotting with a GST antibody indicating that JrCDPK13L-GST was pulled down by JrERF113L-His, but not by the GST-empty vector control. f) Co-immunoprecipitation (Co-IP) assays detect the interaction between JrCDPK13L and JrERF113L in vivo. Total proteins were extracted from *N. benthamiana* leaves co-expressing JrCDPK13L-FLAG and JrERF113L-GFP and immunoprecipitated (IP) with GFP antibody. The co-precipitated JrCDPK13L-FLAG was detected by immunoblotting (IB) with FLAG antibody. Asterisks indicate significant differences compared with the control (two-tailed Student's *t*-test, *P < 0.05, ** *P* < 0.01). JrERF96L was used as a negative control for the interaction assays.

Increasing evidence suggested that ERF family transcription factors as substrate proteins for CDPKs are vital regulators of plant immune responses (Ma et al. 2023). We therefore hypothesized that JrCDPK13L might function in walnut resistance to anthracnose by directly targeting the ERF TFs. To test this hypothesis, we first conducted protein-protein interaction network analysis using CDPKs and ERF TFs, and the results showed a potential interaction relationship between JrCDPK13L and JrERF113L (LOC108989001) (Figure S5a). Subsequently, to verify the interaction between JrCDPK13L and JrERF113L, the Y2H experiment was conducted. The yeast strain Y2HGold transformed with pGBKT7-JrERF113L showed self-activating activities. We then used different concentrations of 3-Amino-1,2,4-triazole (3-AT) to inhibit its self-activation and found that when the concentration of 3-AT reached 50 mM, the self-activation of pGBKT7-JrERF113L disappeared (Figure S5b). The results of Y2H indicated that Y2HGold co-transformed with pGBKT7-JrERF113L and pGADT7-JrCDPK13L grew on SD/-Ade/-His/-Leu/-Trp adding 50 mM 3-AT medium, while control strains transformed with only one plasmid did not grow (Fig. 3a). JrERF96L (*Juglans regia* ethylene-responsive transcription factor ERF096-like, LOC109006752) from the same family as JrERF113L, but did not appear in the protein-protein interaction network between CDPK and ERF families. Therefore, JrERF96L was used as a negative control for subsequent experiments. Coexpression of JrCDPK13-cLUC and JrERF113L-nLUC in *Nicotiana benthamiana* leaves resulted in stronger luminescence than negative control (Fig. 3b, c), indicating that JrCDPK13L interacts with JrERF113L.

The bimolecular fluorescence complementation (BiFC) assays were also conducted to confirm the interaction between JrCDPK13L and JrERF113L. Strong yellow fluorescent protein (YFP) signals were detected in the *N. benthamiana* nucleus coexpressing *JrCDPK13L* and *JrERF113L* (Fig. 3d). Meanwhile, we conducted pull-down assays using reconstructed JrERF113L-HIS fusion proteins with JrCDPK13L-GST and the GST empty vector. After copurification, JrCDPK13L-GST was observed to be pulled down by JrERF113L-HIS (Fig. 3e). Additionally, the co-immunoprecipitation (Co-IP) assays were performed using the transgenic *N. benthamiana* overexpressing both *JrCDPK13L* fuzed to a FLAG tag and *JrERF113L* fuzed to a GFP tag. JrCDPK13L-FLAG proteins were immunoprecipitated by JrERF113L-GFP (Fig. 3f). The above results fully demonstrated the interaction between JrCDPK13L and JrERF113L in vitro and in vivo.

### JrERF113L positively regulates walnut resistance to anthracnose

**Figure 4:**
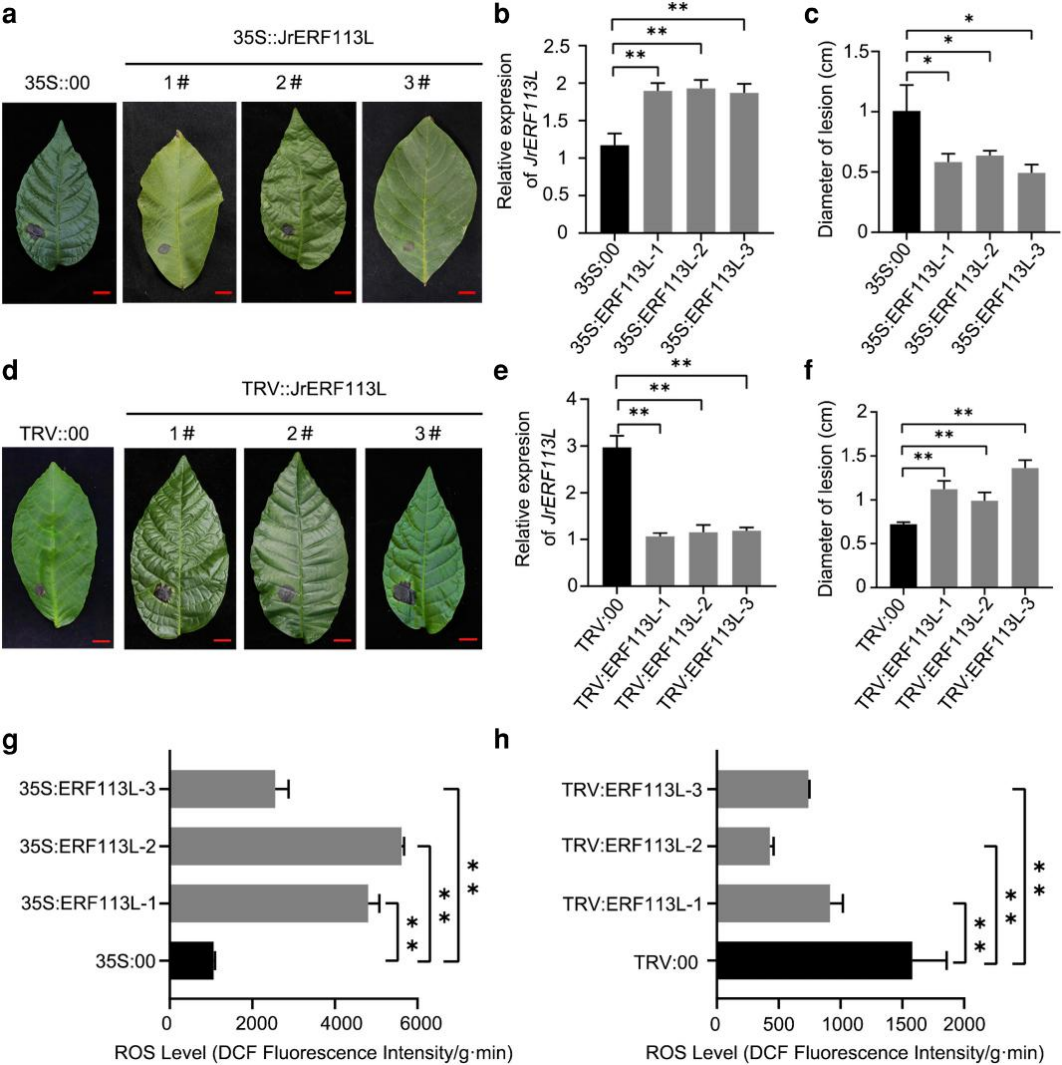
JrERF113L positively regulates walnut resistance to anthracnose. a) Leaf phenotypes of *35S::JrERF113L* and *35S::00* walnut leaves infected with *C. gloeosporioides*. Bars = 1 cm. b) The relative expression levels of *JrERF113L* in *35S::JrERF113L* and *35S::00* walnut leaves. 18S rRNA was amplified as an internal control. c) Diameter of lesions in *35S::JrERF113L* and *35S::00* walnut leaves. d) Leaf phenotypes of *TRV::JrERF113L* and *TRV::00* walnut leaves infected with *C. gloeosporioides*. Bars = 1 cm. e) The relative expression levels of *JrERF113L* in *TRV::JrERF113L* and *TRV::00* walnut leaves. 18S rRNA was amplified as an internal control. f) Diameter of lesions in *TRV::JrERF113L* and *TRV::00* walnut leaves. g) ROS levels in *35S::JrCDPK13L* and *35S::00* walnut leaves. h) ROS levels in *TRV::JrCDPK13L* and *TRV::00* walnut leaves. Data are presented as means ± SD of three biological replicates. Asterisks indicate significant differences compared with the control (two-tailed Student's *t*-test, *P < 0.05, **P < 0.01).

To investigate the regulatory function of JrERF113L in the infection of walnut by *C. gloeosporioides*, the full-length CDS of *JrERF113L* was cloned into the pZP211-3xFlag vector. The constructed plasmid was introduced into “B37” walnut leaves through *Agrobacterium*-mediated vacuum infiltration. The walnut strains overexpressing *JrERF113L* were successfully obtained through PCR amplification and western blot detection (Fig. 4a, Figure S6). qRT-PCR results indicated that *JrERF113L* expression was significantly elevated in the *35S::JrERF113L* transgenic lines compared to the empty vector control (*35S::00*) (Fig. 4b). Then, detached leaves from control (*35S:00*) and *35S::JrERF113L* plants were inoculated with *C. gloeosporioides*. The *35S::JrERF113L* leaves showed reduced disease symptoms (Fig. 4a) and smaller lesion diameters (Fig. 4c) compared to the control, which indicating *JrERF113L* positively regulates walnut resistance to anthracnose.

To further validate the above results, the loss-of-function analysis of *JrERF113L* was performed using VIGS technology. A *JrERF113L*-specific fragment was transformed into walnut to obtain the walnut strains silenced expressing *JrERF113L* (*TRV::JrERF113L*) (Fig. 4d). PCR using primers pTRV1-F/R and pTRV2-F/JrERF113L-R amplified a 324-bp band only in *TRV::JrERF113L* samples and not in the *TRV::00* control (Figure S7). qRT-PCR also confirmed significantly reduced *JrERF113L* expression in silenced plants (Fig. 4e). Upon pathogen challenge, detached leaves from *TRV::JrERF113L* developed more severe symptoms (Fig. 4d) and larger lesions (Fig. 4f) compared to the control. Meanwhile, compared with the control group, the accumulation of ROS in *35S::JrERF113L* leaves was significantly increased, while the content in *TRV::JrERF113L* decreased (Fig. 4g, h). These results demonstrated that *JrERF113L* can enhance walnut resistance to anthracnose.

### JrERF113L binds to the promoter of JrPR5L and promotes its transcription

ERF transcription factors contain one or two AP2/ERF DNA-binding domains, which typically bind to GCC-box (AGCCGCC) or GCCGAC motif in promoter regions to regulate genes involved in plant development, abiotic stress responses, and immunity (Feng et al. 2020). The phylogenetic tree was constructed with JrERF113L, 50 *A. thaliana* ERF TFs, and 6 ERF TFs in other species via MEGA 7. The results of phylogenetic tree analysis revealed that JrERF113L belongs to group VIⅠ and has the highest homology with RAP2-2-like (LOC121263867) from *Juglans microcarpa×Juglans regia* (Figure S8a). The alignment of the amino acid sequences indicated that JrERF113L has a single 858-bp open reading frame (ORF) and encodes a protein with 285 amino acids. As predicted, JrERF113L contained a AP2 domains (YRG motif and RAYD motif), which were conserved at the C-terminus (Figure S8b). Then, we conducted a yeast assay to test whether JrERF113L had transcriptional activation activity. The recombinant plasmids JrERF113L-pGBKT7 and empty plasmid pGBKT7 were transformed into Y2HGold strain, respectively. Compared to the control group, only yeast transformed with JrERF113L-pGBKT7 grew normally on SD media lacking Trp, His, and Ade (SD/-Trp-His-Ade), indicating that JrERF113L had the transcription activation (Figure S9).

**Figure 5:**
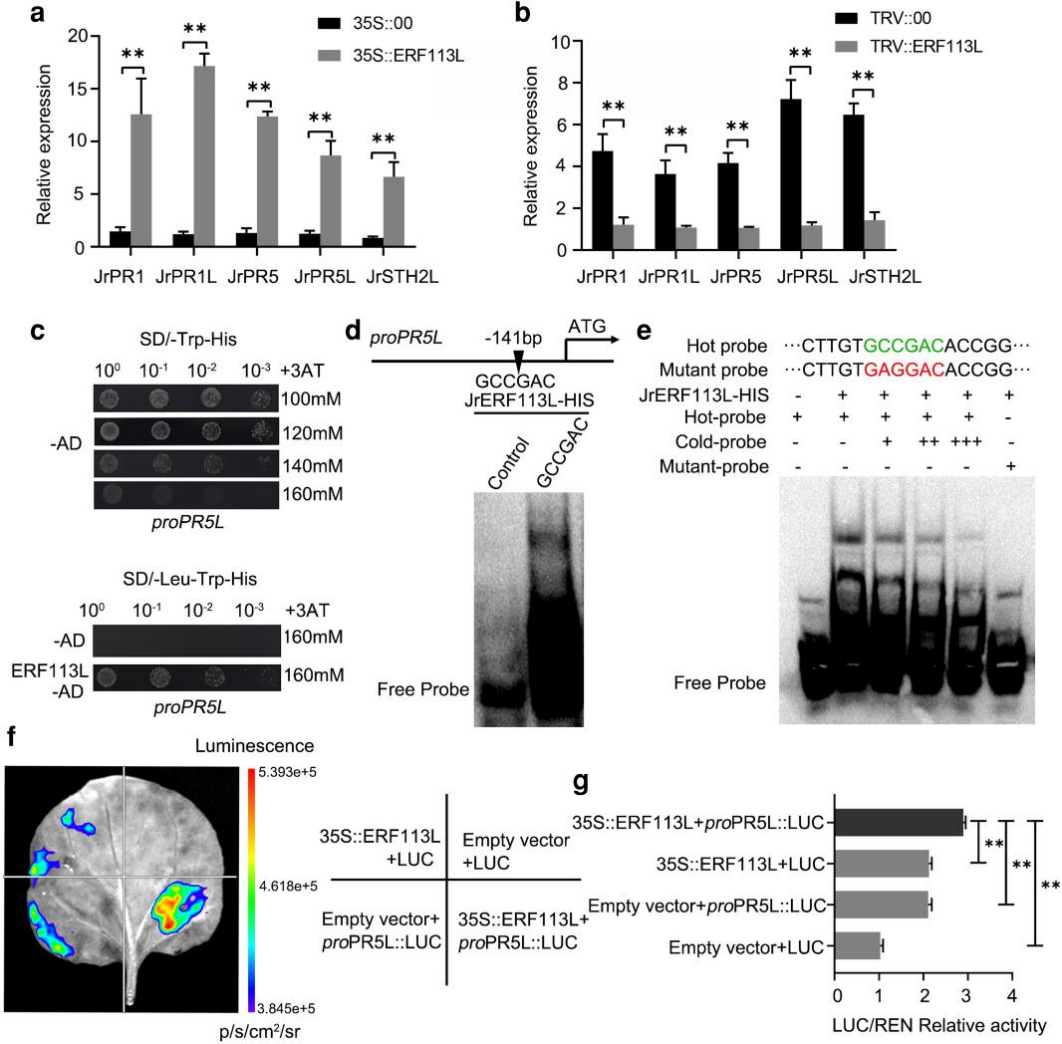
JrERF113L binds to the promoter of JrPR5L and promotes its transcription. a) The relative expression levels of *PR*s gene (*JrPR1*, *JrPR1L*, *JrPR5*, *JrPR5L*, and *JrSTH2L*) in *35S::JrCDPK13L* and *35S::00* walnut leaves. b) The relative expression levels of *PR*s gene in *TRV::JrCDPK13L* and *TRV::00* walnut leaves. c) Yeast one-hybrid (Y1H) assays show that JrERF113L binds to the JrPR5L promoter. The pHIS2 vector containing cis-elements of the JrPR5L promoter was co-transformed with JrERF113L-pGADT7 or empty pGADT7 vector into yeast strain. Transformants were grown on SD/-Leu medium supplemented with different concentrations of 3-AT. d) Electrophoretic mobility shift assays (EMSAs) demonstrate specific binding of JrERF113L to the GCCGAC motif in the JrPR5L promoter. e) Binding specificity verification. Unlabeled wild-type probes effectively competed for binding as cold competitors, whereas biotin-labeled mutant probes (mProbe) failed to bind JrERF113L protein. f) Dual-luciferase reporter assays demonstrate that JrERF113L activates the JrPR5L promoter. g) LUC/REN relative activity in tobacco leaves infected with different vector combinations. Asterisks indicate significant differences compared with the wild-type (WT) control (two-tailed Student's *t*-test, *P < 0.05, **P < 0.01).

An increasing body of evidence suggested that the *PR* genes had been essential mediated factors in the plant immune regulatory network (Mu et al. 2024; Gong et al. 2025). Therefore, we first analyzed the expression levels of the *PR* genes in the *JrERF113L* transgenic walnut strain, and the results showed that the expression of *JrPR1*, *JrPR1L*, *JrPR5*, *JrPR5L*, and *JrSTH2L* were significantly increased in *35S::JrERF113L* and significantly decreased in *TRV:JrERF113L* compared to the control group (Fig. 5a, b). Our previous research has shown that there is a GCCGAC located at 141 bp on the *JrPR5L* promoter (Zhou et al. 2022), which leads us to suspect the binding of JrERF113L to the *JrPR5L* promoter. A Y1H assay showed that JrERF113L binds to the *JrPR5L* promoter (Fig. 5c). To confirm this result, we purified the JrERF113L-His protein and conducted EMSAs. JrERF113L caused a substantial shift of the biotin labeled probe, indicating the presence of the protein-DNA complex (Fig. 5d). When an unlabeled probe as a competitor was added, the binding of JrERF113L to the *GCCGAC motif* was weakened. Meanwhile, no shifted band was observed when a mutated labeled probe (GAGGAC) was used (Fig. 5e). These results demonstrated that JrERF113L bound to the *GCCGAC motif* in the promoter of *JrPR5L*.

Next, we confirmed the effect of JrERF113L on the promoter activity of *JrPR5L* through the transient dual-luciferase assay in *N. benthamiana* leaves. The CDS of *JrERF113L* was inserted into the pGreenII62-SK vector as an effector. The promoter sequence of *JrPR5L* was fuzed to the pGreenII 0800-LUC vector as a LUC reporter gene (Figure S10). Luminescence detection exhibited that the luminescence intensity of the coexpression of *35S:JrERF113L* with *proJrPR5L::LUC* was significantly higher than that of the individual expression of *proJrPR5L::LUC* (Fig. 5f, g). These results indicate that JrERF113L activates the transcriptional activity of *JrPR5L* by binding to *GCCGAC motif*.

### JrCDPK13L phosphorylates JrERF113L at specific sites to enhance its stability

**Figure 6:**
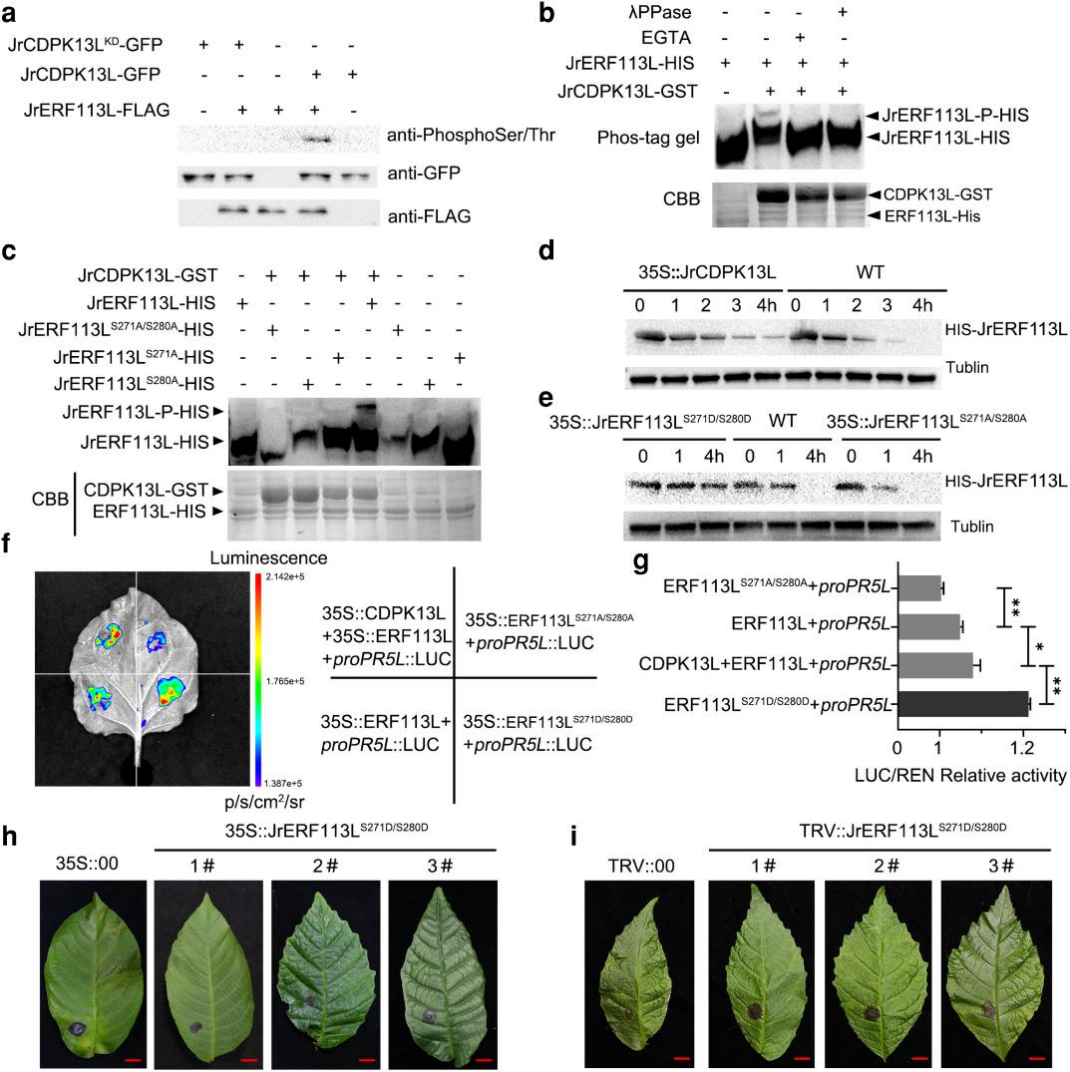
JrCDPK13L phosphorylates JrERF113L at Ser271/Ser280 to enhance its stability, transcriptional activity, and disease resistance function. a) In vivo phosphorylation assays demonstrate that JrCDPK13L phosphorylates JrERF113L. Proteins (JrCDPK13L-GFP and JrERF113L-FLAG) were immunoprecipitated from transgenic tobacco leaf using an anti-GFP and anti-FLAG antibody and separated using SDS-PAGE for immunoblot analysis. An anti-phosphoSer/Thr antibody was used to detect JrERF113L phosphorylation. b) Phos-tag mobility shift assays demonstrate that JrCDPK13L phosphorylates JrERF113L in vitro. Proteins were separated on a phostag gel and an immunoblot analysis was performed using an anti-His antibody to detect JrERF113L. The slowly migrating band on the phos-tag gel represents phosphorylated JrERF113L-His (upper panel). CBB staining served as the loading control (bottom panel). c) Phos-tag mobility shift assays identify Ser271 and Ser280 as phosphorylation sites in JrERF113L. JrERF113L variants harboring alanine substitutions at S271 and/or S280 (JrERF113L^S271A^, JrERF113L^S280A^, JrERF113L^S271A/S280A^). Reaction products were analyzed by Phos-tag and regular SDS-PAGE, followed by immunoblotting with anti-His antibody (upper panel). CBB staining served as the loading control (bottom panel). d) In vitro cell-free degradation assays show that JrCDPK13L-mediated phosphorylation stabilizes JrERF113L. Recombinant JrERF113L-His protein was incubated with total protein extracts from empty vector or 35S:: JrCDPK13L transgenic tobacco leaves at indicated time points, followed by immunoblot analysis with anti-His antibody. Tubulin served as the loading control. e) In vitro cell-free degradation assays compare the stability of phosphorylation-defective (JrERF113L^S271A/S280A^) and phosphomimetic (JrERF113L ^S271D/S280D^) variants. Purified proteins were incubated with total protein extracts from *35S::JrERF113L ^S271D/S280D^* or *35S::JrERF113L^S271A/S280A^* tobacco leaves, followed by immunoblot analysis with anti-His antibody. Tubulin served as the loading control. f) Representative luminescence images of *N. benthamiana* leaves co-infiltrated with the reporter (*proJrPR5L::LUC*) and different effector constructs. g) Quantitative analysis of LUC/REN relative activity. h) Leaf phenotypes of *35S::JrERF113L^S271D/S280D^* walnut leaves infected with *C. gloeosporioides*. Bars = 1 cm. i) Leaf phenotypes of *TRV::JrERF113L^S271D/S280D^* walnut leaves infected with *C. gloeosporioides*. Bars = 1 cm.

The interaction between JrCDPK13L and JrERF113L initiated the question of whether JrERF113L was a direct substrate of JrCDPK13L.We first employed an in vivo phosphorylation assay to test this possibility through transiently infiltrated *N. benthamiana* leaves with JrCDPK13L-GFP, JrERF113L-FLAG, and kinase-inactive mutant ofJrCDPK13L (JrCDPK13L^KD^-GFP). Immunoblotting of total protein extracts using anti-phosphoserine/threonine-specific antibodies revealed that the phosphorylation signal was only detected in leaves coexpressing *JrCDPK13L* and *JrERF113L*, confirming the phosphorylation of JrERF113L depended on JrCDPK13L (Fig. 6a). Meanwhile, the recombinant GST-fuzed JrCDPK13L and HIS-fuzed JrERF113Lwere used for in vitro phosphorylation validation. We observed a phosphorylation signal in the presence of JrERF113L-HIS and JrCDPK13L-GST compared to the negative control, which was significantly reduced by λ-phosphatase and EGTA (Fig. 6b). These results indicated that JrCDPK13L phosphorylates JrERF113L.

Since the GPS 6.0 database (http://gps.biocuckoo.org/) and NetPhos-3.1 (https://services.healthtech.dtu.dk/services/NetPhos-3.1/) lacked specific data for CDPK kinases, we used related kinase models (AGC and CaMK) to predict phosphorylation sites in the JrERF113L protein (Harmon et al. 2001; Zhang et al. 2023; Goher et al. 2025). Based on further screening of the phosphorylation site prediction results, we found two possible phosphorylation sites at serine residues 271 and 280 (Figure S11). To verify whether S271 and S280 are potential phosphorylation sites, we generated and purified JrERF113L variants with alanine mutations at S271 or/and S280 in *Escherichia coli* to form JrERF113L^S271A^, JrERF113L^S280A^, and JrERF113L^S271A/S280A^, respectively. We then checked the phosphorylation level of JrERF113L^S271A^, JrERF113L^S280A^, and JrERF113L^S271A/S280A^ using in vitro phosphorylation assay. Immunoblot analysis showed that the phosphorylation signals of JrERF113L^S271A^ and JrERF113L^S280A^ were weaker than that of JrERF113L, and the phosphorylation signal disappeared when we used JrERF113L^S271A/S280A^ as the substrate (Fig. 6c). Collectively, these results shown that JrCDPK13L phosphorylated JrERF113L at Ser271/Ser280.

Next, an in vitro cell-free degradation assay was performed to reveal the effect of JrCDPK13L-dependent phosphorylation of JrERF113L on its protein stability. In contrast to that from the empty vector, JrERF113L degradation was strongly weakened when recombinant JrERF113L was incubated with total proteins extracted from the overexpressed *JrCDPK13L N. benthamiana* leaves (Fig. 6d). To further validate the result, we generated the simulated phosphorylation mutant of JrERF113L to form JrERF113L^S271D/S280D^ and compared the protein stability of JrERF113L in the WT, JrERF113L^S271A/S280A^ and JrERF113L^S271D/S280D^. The results suggested that JrERF113L^S271D/S280D^ attenuated the protein degradation of JrERF113L compared with WT. On the contrary, JrERF113L^S271A/S280A^ promoted the protein degradation of JrERF113L (Fig. 6e). Meanwhile, the pull-down assay showed that the interaction intensity between JrCDPK13L and JrERF113L was enhanced using reconstructed JrERF113L^S271D/S280D^-HIS fusion proteins with JrCDPK13L-GST (Fig. 3e). These results demonstrated that the phosphorylation of JrERF113L, which was dependent on JrCDPK13L, affected JrERF113L degradation and the intensity of their interaction.

### Phosphorylation of JrERF113L promotes transcription of *JrPR5L* and walnut disease resistance

Since the phosphorylation of JrERF113L by JrCDPK13L alters protein abundance of JrERF113L, the regulatory effect of JrERF113L on *JrPR5L* should be strengthened. Firstly, we investigated the effect of phosphorylation of JrERF113 by JrCDPK13L on the binding activity of JrERF113L and *JrPR5L* promoter using EMSA assay. The result showed a marked increase in JrERF113L binding to the *JrPR5L* promoter following pre-incubation with JrCDPK13L relative to the non-phosphorylated control (Figure S12). In addition, we conducted the dual-luciferase assay using pGreenII62-SKvector harboring JrERF113L, JrERF113L^S271A/S280A^, JrERF113L^S271D/S280D^, and coexpressed JrCDPK13L and JrERF113L as effectors, respectively (Figure S13). We observed that when coexpressing with *ProJrPR5L::LUC*, the *35S::JrERF113L^S271D/S280D^* group had the highest LUC activity, followed by the group coexpressed *35S::JrCDPK13L* and *35S::JrERF113L*, while the *35S::JrERF113L^S271A/S280A^* group had the lowest activity (Fig. 6f, g). This observation suggested that *C. gloeosporioides* induced *JrPR5L* transcript levels through JrERF113L, which was enhanced by JrERF113L phosphorylation by JrCDPK13L.

Meanwhile, we investigated the effect of phosphorylation sites (Ser271/Ser280) of JrERF113L by JrCDPK13L on the resistance to anthracnose function of walnut. PCR and western blot verification had confirmed that *JrERF113L^S271D/S280D^* had been successfully transferred into walnut leaves (*35S::JrERF113L^S271D/S280D^*) (Figure S14). After inoculation, detached leaves from *35S:JrERF113L^S271D/S280D^* displayed reduced disease symptoms (Fig. 6h) and smaller lesion diameters (Figure S17a) compared to the control. Correspondingly, the ROS content and *PRs* gene expression of *35S:JrERF113L^S271D/S280D^* leaves were significantly higher than those in *35S: 00* (Figure S15). Meanwhile, the walnut strain expressing *JrERF113L^S271D/S280D^* in silence were obtained using VIGS technology (Figure S16). After inoculation, detached leaves from *TRV:JrERF113L^S271D/S280D^* displayed increased disease symptoms (Fig. 6i) and larger lesion diameters (Figure S17b) compared to the control. These results indicated that the phosphorylation of JrERF113L by JrCDPK13L at Ser271/Ser280, strengthening transcriptional activation of *JrPR5L* and resulting in walnut resistance to anthracnose.

## Discussion

As a crucial second messenger, Ca^2+^ signaling plays an important role in plant immunity, involving changes in intracellular Ca^2+^ concentration and binding to Ca^2+^ sensors to activate downstream immune responses (Wang et al. 2024a; Zhang et al. 2024). Among various Ca^2+^ sensors, CDPKs have been widely reported due to their ability to integrate sensing and response activities (Poovaiah et al. 2013). In *Arabidopsis*, AtCPK4/5/6/11 localize in both nucleus and cytosol and function as positive regulators of flg22-induced PTI responses and ROS generation (Boudsocq et al. 2010). In addition, AtCPK28 serves as a negative regulator of BOTRYTIS INDUCED KINASE 1 (BIK1)-regulated immunity in response to PAMP-induced Ca^2+^ bursts (Monaghan et al. 2015). Meanwhile, phosphorylation of AtCPK28 at Ser318 induces a conformational change, enabling AtCPK28 to be activated under low Ca^2+^ concentrations and thus rapidly respond to immune signals (Bredow et al. 2021). TaCDPK7 mediates ROS accumulation and promotes the expression of PR genes *TaPR1*, *TaPR2*, *TaPR5*, thereby enhancing wheat defense against *Puccinia striiformis tritici* (*Pst*) (Goher et al. 2024). Here, we identified a Ca^2+^ -dependent protein kinase, JrCDPK13L, that responds to *C. gloeosporioides* infection in walnut. Through disease resistance evaluation of transgenic lines, JrCDPK13L acts as a positive regulator of plant immunity by promoting walnut resistance to anthracnose, and its expression is transcriptionally induced upon infection, which also boosts ROS burst and *PRs* gene expression. Therefore, elucidating how JrCDPK13L contributes to walnut resistance to anthracnose is of considerable interest.

Biochemical and genetic evidence suggests that CDPKs act as central regulators that trigger various downstream responses in plant immunity signaling networks by phosphorylating diverse substrates including transcription factors, metabolic enzymes, and ion channels (Asano et al. 2012; Monaghan et al. 2014). In rice (*Oryza sativa*), OsCPK17 interacts with and phosphorylates OsRLCK176 at Ser83, which inhibits the ubiquitination of OsRLCK176 by OsPUB12 and maintains rice immune homeostasis (Mou et al. 2024). Additionally, OsCPK18 acts as an upstream kinase phosphorylates and activates OsMAPK5 at Thr-14 and Thr-32, which restrains defense gene expression and negatively mediates rice resistance to blast (Xie et al. 2014). In tomato (*Solanum lycopersicum*), LeCDPK2 phosphorylates the 1-aminocyclopropane-1-carboxylic acid (ACC) synthase (ACS) LeACS2 at Ser-460 in response to a wounding signal (Kamiyoshihara et al. 2010). In *Arabidopsis*, CDPK5 phosphorylates calmodulin-binding transcription activator 3 (CAMTA3) at Ser964 and contributes to its destabilization, thus controlling the onset of autoimmunity in *exo70B1* (Liu et al. 2024). The latest research shows that CPK4 and CPK11, activated by *Sclerotinia sclerotiorum*, phosphorylate WRKY8 and release its inhibition of RLP23 to balance the trade-off between growth and immunity (Ren et al. 2024). In this study, we show that JrCDPK13L interacts with JrERF113L and phosphorylates it at Ser271/Ser280, thereby promoting JrERF113L stability. However, the effects of JrCDPK13L-mediated phosphorylation of JrERF113L on downstream signaling and walnut resistance to anthracnose deserve further investigation.

The roles of ERF transcription factors as a bridge connecting upstream signals to downstream functional genes in plant immunity signaling networks have been extensively documented (Licausi et al. 2013; Feng et al. 2020). In soybean (*Glycine max*), both GmERF5 and GmERF113 have been shown to enhance soybean resistance to *Phytophthora sojae* (Dong et al. 2015; Zhao et al. 2017). In wild grapevine (*Vitis pseudoreticulata*), VpERF2 and VpERF3 improve resistance to both the oomycete *Phytopthtora parasitica* and the bacterial pathogen *Ralstonia solanacearum* (Zhu et al. 2013). In this study, we found that overexpression of *JrERF113L* in walnut leaves promotes disease resistance, while silencing *JrERF113L* expression increases susceptibility, indicating that JrERF113L acts as a positive regulator of walnut resistance to anthracnose. ERF proteins specifically bind to cis-elements of GCC-box (AGCCGCC), DRE/CRT (A/GCCGAC) in promoter regions of pathogen induced genes (Catinot et al. 2015). AtERF9 as a transcription repressor inhibits *AtPDF1.2* expression by binding to the GCC-box of the *PATHGEN-INDUCIBE PLANT DEFENSIN* (*PDF1.2*) gene (Maruyama et al. 2013). MdERF114 enhances lignin accumulation and root resistance against *Fusarium solani* by directly binding to the GCC-box in the *MdPRX63* promoter and activating its expression (Liu et al. 2023). In our study, it is shown that JrERF113L directly binds to the GCCGAC motif in the *JrPR5L* promoter and induces its expression, thereby conferring walnut resistance to anthracnose. This finding is consistent with our previous report on the regulation of *JrPR5L* by another ERF TF JrPTI5L (Zhou et al. 2022), and raises the question of whether the mechanism by which ERFs regulate walnut resistance to anthracnose is conserved.

Recently, with the advancement and integration of walnut genome sequencing, gene editing, and molecular biology technologies (Yan et al. 2021; Song et al. 2025), the interaction mechanisms between walnut and *C. gloeosporioides* have been widely investigated. From the perspective of *C. gloeosporioides* infection, it has been found that *C. gloeosporioides* secretes the effector proteins in a stage-specific manner, while the walnut host exhibits genotype-specific defense responses, with resistant lines displaying a more comprehensive response (Li et al. 2024). Meanwhile, multiple omics studies have been fully utilized to explore a series of key genes, proteins, and taxonomic compounds associated with walnut resistance to anthracnose (Fang et al. 2021; Xu et al. 2022; Gong et al. 2025). Furthermore, multiple molecular modules regulating walnut resistance to anthracnose have also been extensively studied. The long non-coding RNA lncRNA109897 and its target gene *JrCCR4* form a positive feedback regulatory loop with JrTLP1b to enhance walnut resistance to *C. gloeosporioides* (Feng et al. 2021; Zhou et al. 2023). Both the JrPHL8-JrWRKY4-JrSTH2L module and JrWRKY21-JrPTI5L-JrPR5L module have been confirmed to positively regulate walnut resistance to anthracnose (Zhou et al. 2022; Mu et al. 2024). However, research on walnut disease resistance genes has largely remained at the transcriptional protein levels, and little is known about the role of post-translational mechanisms. Here, we found that JrCDPK13L induced phosphorylation of JrERF113L promotes the transcription of downstream gene *JrPR5L*, which was also confirmed using simulated phosphorylation mutant JrERF113L^S271D/S280D^. Meanwhile, walnut leaves overexpressing *JrERF113L^S271D/S280D^* exhibited enhanced disease resistance.

**Figure 7:**
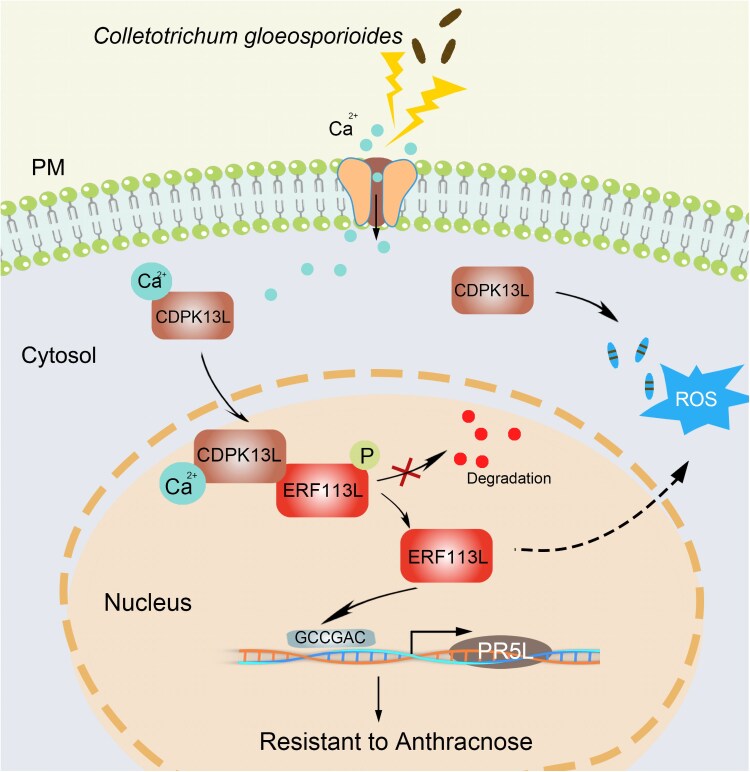
A working model showing the mechanism of the JrCDPK13L- JrERF113L module in regulating walnut resistance to *C. gloeosporioides*. *C. gloeosporioides* triggered Ca^2+^ signal activates JrCDPK13L and improves its expression. JrCDPK13L interacts with and phosphorylates JrERF113L and enhances the protein stability of JrERF113L, thereby enhancing the expression levels of JrPR5L and promoting the accumulation of ROS to promote walnut resistance to *C. gloeosporioides*.

In conclusion, our findings establish the JrCDPK13L-JrERF113L module as a pivotal component of the response mechanism to *C. gloeosporioides* infection. We demonstrate that JrCDPK13L acts as a Ca^2+^ sensor that is integral to the Ca^2+^-dependent defense response pathway against *C. gloeosporioides*. Our findings reveal that JrCDPK13L interacts with and phosphorylates JrERF113L, thereby regulating *JrPR5L* expression and ROS accumulation, thereby in turn enhances walnut resistance to *C. gloeosporioides* (Fig. 7). This study uncovers the molecular and biological functions of JrCDPK13L and delves into the mechanisms of the JrCDPK13L-JrERF113L module during *C. gloeosporioides* infection, providing profound insights into the complex molecular regulatory network that governs walnut responses to this pathogen.

## Materials and methods

### Plant materials and growth conditions

Leaves from the highly anthracnose-susceptible walnut cultivar “B37” (designated L37) and the highly resistant cultivar “4–23”(designated L423) served as controls for transient overexpression (35S::00) and virus-induced gene silencing (TRV::00), respectively. *Nicotiana benthamiana* plants were maintained in a growth chamber at 25 °C under a 16/8-hour light/dark cycle with a light intensity of 120 µmol m^−2^ s^−1^. For leaf inoculations, the *C. gloeosporioides* strain “m9” (GenBank accession no. GU597322) was used.

### Pathogen inoculation

The pathogen used in this study was the *C. gloeosporioides* strain “m9”(GenBank accession no. GU597322). The strain was maintained on potato dextrose agar (PDA) plates. For plant inoculation, the fungus was subcultured onto fresh PDA plates and incubated at 22 °C for 7 to 12 d to promote robust mycelial growth and condition. A sterile cork borer was used to cut 5-mm diameter agar plugs from the actively growing edge of the fungal colonies. For walnut leaf inoculation, the leaves were first gently wounded at the inoculation site using a sterile blade to create minor abrasions. The fungal agar plug was then placed upside down (with the mycelial side facing the leaf) onto the wounded area.

### Measurement of Ca^2+^ influx using NMT

Leaf tissues around the lesions were excised from target leaves at 48 h post-inoculation with *C. gloeosporioides*. The mesophyll was exposed by removing the abaxial epidermis, then immersed in test solution (0.5 mM CaCl_2_, 0.2 mM MES, pH 5.8) for 3 h to measure the Ca^2+^ influx rate in the mesophyll. Each sample was recorded for 5 min under illumination consistent with the cultivation light intensity throughout the experiment.

### Transcriptome data analysis

Transcriptome data were utilized to conduct homologous gene alignment analysis via the NCBI (https://www.ncbi.nlm.nih.gov/) database. This was followed by a comprehensive analysis of the protein sequences encoded by candidate genes employing the online tool SMART (http://smart.embl-heidelberg.de/) and DNAMAN software, to predict their functional domains and signal peptide characteristics. A phylogenetic tree was subsequently constructed using MEGA7 software. To validate the bioinformatics analysis, total RNA was extracted from walnut leaves following the method described by (Zhou et al. 2023). Reverse transcription was performed using HiScript III RT SuperMix for qPCR (+gDNA wiper) (Vazyme Biotech Co., Ltd., Nanjing).

### qRT-PCR

Gene expression was quantified by quantitative real-time PCR (qRT-PCR) using 2× SYBR qPCR Master Mix. The internal reference is 18S rRNA, and relative expression levels of target genes were determined using the 2^−ΔΔCT^ method implemented in IQ5 2.0 software. The primers for RT-qPCR were synthesized by Sangon Biotech (Table S2, http://www.sangon.com).

### Virus-induced gene silencing (VIGS) assay

For VIGS construct preparation, specific CDS of JrCDPK13L and JrERF113L were amplified from cDNA of the resistant walnut cultivar “4–23” and cloned into the pTRV2 vector to generate pTRV2-JrCDPK13L and pTRV2-JrERF113L. Empty vectors (pTRV1, pTRV2) and recombinant constructs were introduced into *Agrobacterium tumefaciens* GV3101-pSoup. Bacterial suspensions were vacuum- infiltrated into leaves of “L423” seedlings as described Zhou et al. (2023). The experiment included three biological replicates with three leaves sampled per replicate for phenotypic assessment.

### Transient overexpression assay

The CDS of JrCDPK13L, JrERF113L, and the phosphomimetic variant JrERF113L^S271D/S280D^ were cloned into the pzp211-3xflag vector. Constructs and the empty vector were transformed into *A. tumefaciens* GV3101. Leaves of the susceptible cultivar “B-37” were infiltrated with bacterial suspensions as in the VIGS assay. Each treatment consisted of three biological replicates, with three leaves collected per replicate. After 2 d of dark incubation, plants were inoculated with pathogen. Disease symptoms were recorded and tissues were harvested at 4d post-inoculation.

### Detection of reactive oxygen species in plant tissues

Walnut leaves from experimental and control groups were inoculated with *C. gloeosporioides*. Leaf tissues were flash-frozen in liquid nitrogen and ground into fine powder. Then, 0.1 g of powdered tissue was used for reactive oxygen species (ROS) quantification using a fluorescence-based assay kit (Suzhou Grace Biotechnology Co., Ltd.). Each sample was homogenized in 1 mL of ice-cold extraction buffer and centrifuged at 12,000 × *g* for 10 min at 4 °C. The resulting supernatant was collected and incubated with the fluorescent probe DCFH-DA at 37 °C in the dark for 30 min. Fluorescence intensity was measured using a microplate reader with excitation and emission wavelengths set at 488 and 525 nm.

### Transcriptional activation assay

The CDS of JrERF113L was cloned into the pGBKT7 vector to generate the recombinant plasmid pGBKT7-JrERF113L, which was subsequently transformed into E. coli DH5α competent cells. The plasmid was extracted using the 5fz High Q Plasmid Auto Extraction Kit (Kangma-Healthcode Biotech, Shanghai) and transformed into the yeast Y2HGold competent cells (Weidi Biotech, Shanghai). Yeast cells carrying the empty pGBKT7 vector served as the negative control. Transformed yeast cells were plated on SD/-Trp medium for initial growth. Subsequently, successfully transformed colonies were transferred to selective SD/-Trp/-His/-Ade medium and incubated at 28 °C for 3 d to evaluate the transcriptional activation activity of JrERF113L. All primers used in this experiment are listed in Table S3.

### Yeast two-hybrid (Y2H) assay

The CDS of JrCDPK13L was cloned into pGBKT7, and the CDS of JrERF113L was cloned into pGADT7 to generate recombinant expression vectors. These constructs were co-transformed into Y2HGold yeast competent cells. Yeast colonies were grown on SD/-Trp/-Leu and SD/-Trp/-Leu/-His/-Ade media for 5 d. Subsequent colony growth on the latter medium demonstrated potential protein-protein interactions. Primers used for amplification are listed in Table S3.

### Bimolecular fluorescence complementation (BiFC) assay

In order to construct the recombinant vector, we connected the CDS of *JrCDPK13L* to 35S-pspyCe-YFP, the CDS of *JrERF96L* to 35S-pspyNe-YFP, and the CDS of *JrERF113L* to 35S-pspyNe-YFP. The recombinant vectors were then transformed into *Agrobacterium* GV3101 (Weidi Biotechnology, Shanghai, China), which were co-infiltrated into leaves of 4-wk-old *N. benthamiana*. Fluorescent signals were observed 24–48 h post-infiltration using a Zeiss LSM 880 laser scanning confocal microscope. Primers used for the amplification are listed in Table S3.

### Pull-down assay

The CDS of JrCDPK13L was inserted into the pGEX-4T-1 vector containing a GST-tag sequence, and the CDS of JrERF113L was inserted into the pET-32a vector containing a His-tag sequence. The resulting recombinant plasmids were transformed into E. coli BL21 CodonPlus (DE3) for protein expression and purification. The interaction was subsequently verified by Western blotting. Primers used for amplification are listed in Table S3.

### Yeast one-hybrid assay

The coding sequence of JrERF113L was fuzed to the pGADT7 vector, while a promoter fragment of JrPR5L was inserted into the pHIS2 vector. The optimal concentration of 3-Amino-1,2,4-triazole (3-AT) required for screening the recombinant JrPR5L-pHIS2 construct was determined on SD/-Trp/-His medium. Both constructs were subsequently co-transformed into yeast cells and plated on SD/-Trp/-His/-Leu medium. After 5 d of incubation, protein-DNA interaction was assessed based on yeast growth. All amplification primers are listed in Table S3.

### Electrophoretic mobility shift assay

The recombinant plasmid JrERF113L-pET32a was transformed into E. coli BL21 CodonPlus (DE3) competent cells. The transformed cells were cultured and then lysed by sonication, followed by purification of the recombinant His-tagged JrERF113L protein. Protein-DNA binding reactions were performed using the LightShift Chemiluminescent EMSA Kit (Beyotime). Biotin-labeled probes used in this assay are listed in Table S4. A mutant probe carrying three nucleotide substitutions was included as a specificity control.

### Luciferase complementation assay (LCA)

The CDS of JrERF113L was cloned into the pSPYNE vector, and the CDS of JrCDPK13L was cloned into the pSPYCE vector. The recombinant plasmids were transformed into Agrobacterium tumefaciens strain GV3101-pSoup. The agrobacteria were cultured, resuspended, and used to infiltrate *N. benthamiana* leaves for transient expression. Luminescence from firefly and renilla luciferase was detected using a live imaging system. Primers used are listed in Table S3.

### Dual-luciferase reporter assay

The CDS sequences of JrCDPK13L, JrERF113L, and JrERF113L^S271D/S280D^ were cloned into the pGreenII-62-SK vector. The promoter sequence of *JrPR5L* was inserted into the pGreenII-0800-LUC vector. These recombinant plasmids were transformed into *A. tumefaciens* GV3101. After culture and resuspension, the bacterial suspensions were used to infiltrate *N. benthamiana* leaves for transient expression. Enzymatic activities of firefly and renilla luciferase were measured using a live imaging system. Primers used are listed in Table S3.

### In vitro protein degradation assay in plants

Total protein was extracted from *N. benthamiana* leaves using Plant RIPA Lysis Buffer (Beyotime). Prokaryotically expressed JrERF113L-His protein was mixed with plant total protein (from WT or 35S::JrCDPK13L-FLAG leaves) at a 1:3 ratio and incubated in a metal bath at 23 °C. Samples were taken at 0, 1, 2, 3 and 4 h. Protein levels of JrERF113L-His were examined by Western blotting using an anti-His antibody. Primers used are listed in Table S3.

### Co-immunoprecipitation assay

Total protein was extracted from *N. benthamiana* leaves transiently expressing the target genes using Plant Western and IP lysis buffer. The lysate was pre-incubated with Protein A+G magnetic beads. A 500 µL aliquot of the sample was incubated with GFP antibody at 4 °C overnight, followed by incubation with the beads at room temperature for 2 h with gentle rotation. The bead-bound complexes were collected using a magnetic stand, and the supernatant was removed. The complexes were eluted by boiling in 1× SDS-PAGE loading buffer. The resulting samples were subjected to immunoblotting. Primers used are listed in Table S3.

### In vivo phosphorylation assay

The CDS of JrCDPK13L was cloned into the pHB-GFP vector, and the CDS of JrERF113L was inserted into the pZP211-3×FLAG vector. Each construct was transformed into *A.tumefaciens* GV3101. Bacterial cultures were resuspended and co-infiltrated into *N. benthamiana* leaves. Total protein was extracted using Plant Western and IP lysis buffer. The samples were incubated with GFP antibody and Protein A+G magnetic beads at 4 °C overnight, followed by incubation at room temperature for 2 h. SDS-PAGE loading buffer was added, and the mixture was heated at 99 °C for 5 min. Proteins were separated by 12% SDS-PAGE, and phosphorylation of JrERF113L was detected using a pan-phosphoserine/threonine antibody. Primers are listed in Table S3.

### In vitro phosphorylation assay

Purified JrCDPK13L-GST and JrERF113L-His proteins were mixed in kinase reaction buffer and incubated at 30 °C for 1.5 h. The reaction was terminated by adding SDS-PAGE loading buffer and heating at 99 °C for 7 min. Proteins were separated by 8% SDS-PAGE containing Phos-tag, and phosphorylation of JrERF113L was detected using an anti-His antibody. Primers used are listed in Table S3.

## Supplementary Material

kiag494_Supplementary_Data

## Data Availability

The data underlying this article are available in the article and in its online Supplementary material.
