# Supplementary material for: JrCDPK13L-mediated phosphorylation of JrERF113L promotes walnut resistance to Colletotrichum gloeosporioides
Source: Plant Physiol. 2026 Jul 13;201(3):kiag494. doi: 10.1093/plphys/kiag494 (PMC13418355; doi:10.1093/plphys/kiag494)
Supplement: kiag494_Supplementary_Data [file kiag494_supplementary_data.zip › Supplymentary Figure.pdf]

A

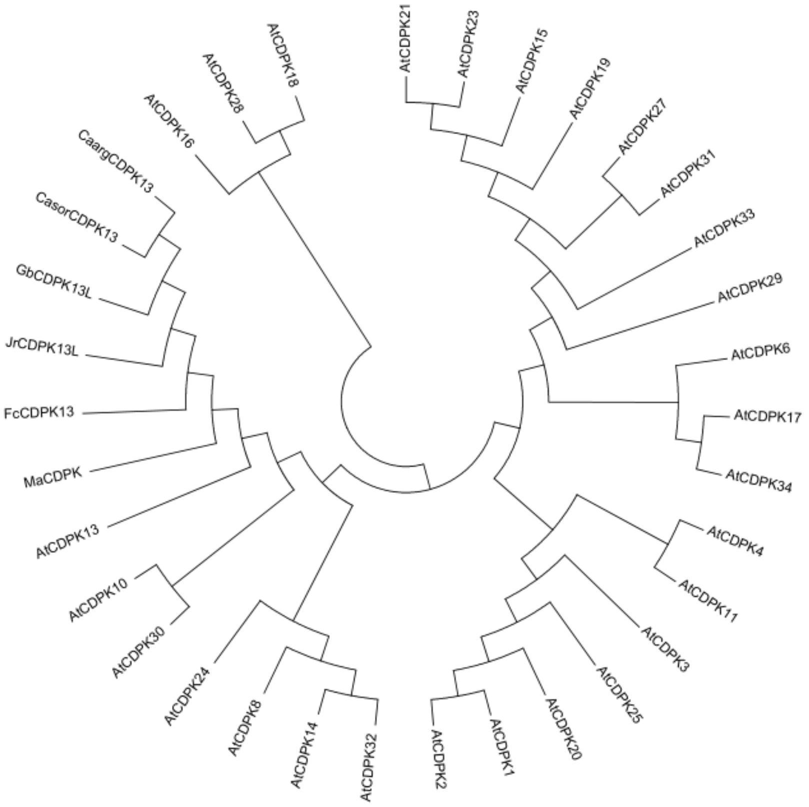
AtCDPK18
AtCDPK28
AtCDPK16
CaargCDPK13
CasorCDPK13
GbCDPK13L
JrCDPK13L
FcCDPK13
MaCDPK
AtCDPK13
AtCDPK10
AtCDPK30
AtCDPK24
AtCDPK8
AtCDPK14
AtCDPK32
AtCDPK2
AtCDPK1
AtCDPK20
AtCDPK25
AtCDPK3
AtCDPK11
AtCDPK4
AtCDPK34
AtCDPK17
AtCDPK6
AtCDPK29
AtCDPK33
AtCDPK31
AtCDPK27
AtCDPK19
AtCDPK15
AtCDPK23
AtCDPK21

B

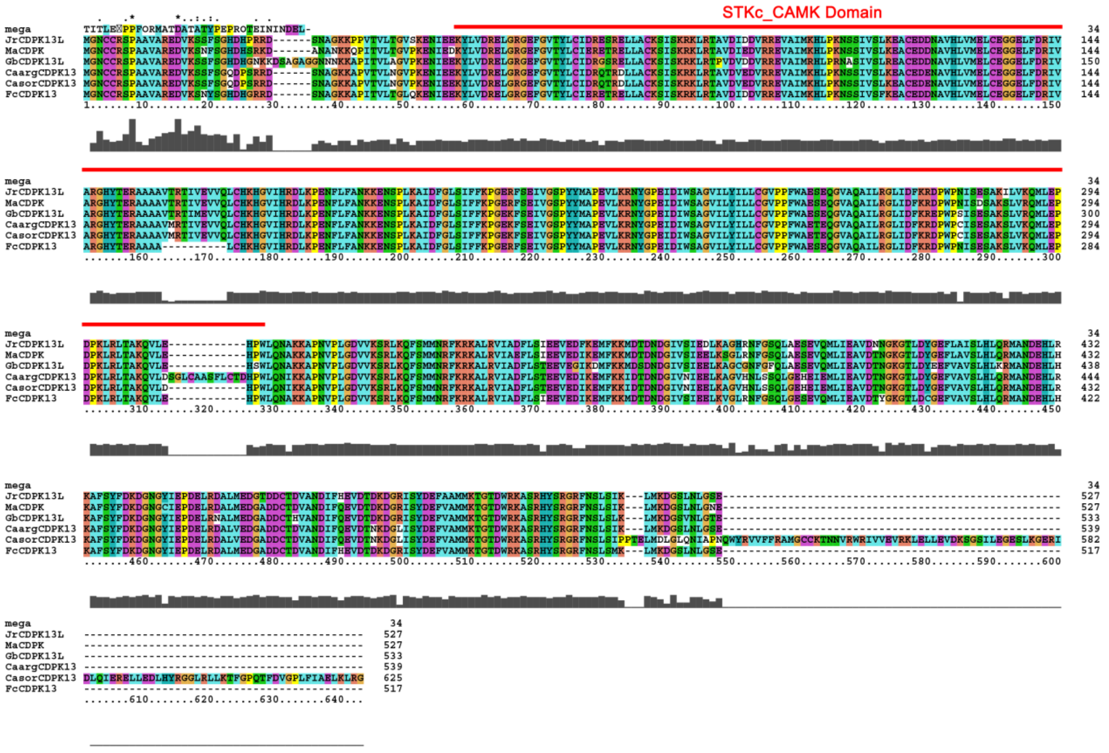
STKc_CAMK Domain
mega
JrCDPK13L
MaCDPK
GbCDPK13L
CaargCDPK13
CasorCDPK13
FcCDPK13

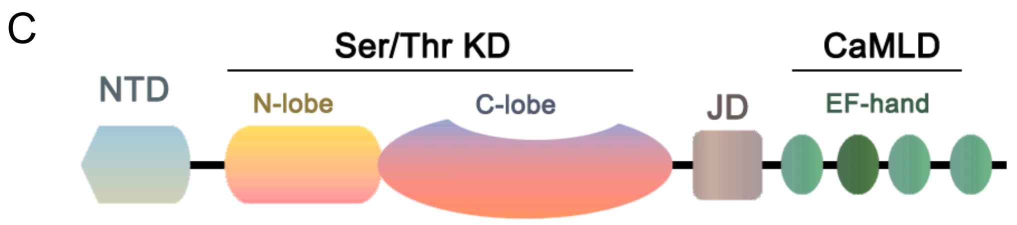

Supplementary Figure S1. Bioinformatic Characterization of JrCDPK13L. (A) A phylogenetic tree constructed using the maximum likelihood method with 28 CDPK family proteins and 5 homologous proteins of JrCDPK13L. (B) The amino acid sequences of six CDPKs, including the STKc-CAMK domain. (C) Analysis of the JrCDPK13L protein EF-hand domain using the PROSITE database.

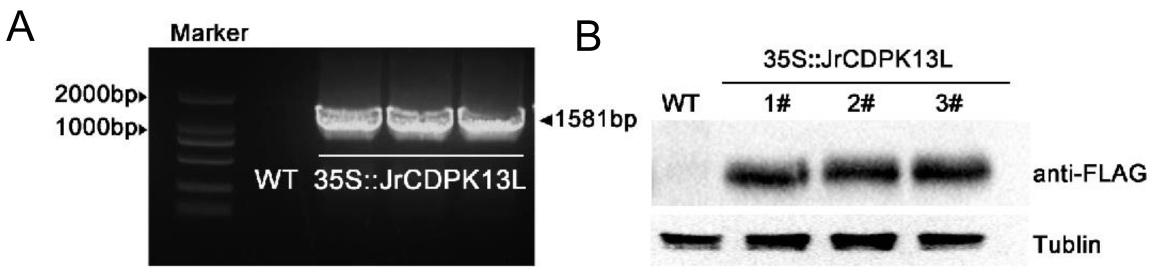

Supplementary Figure S2. The presence of the transgene in *35S::JrCDPK13L* walnut leaves was detected by PCR amplification (A) and western-blot with FLAG antibody (B).

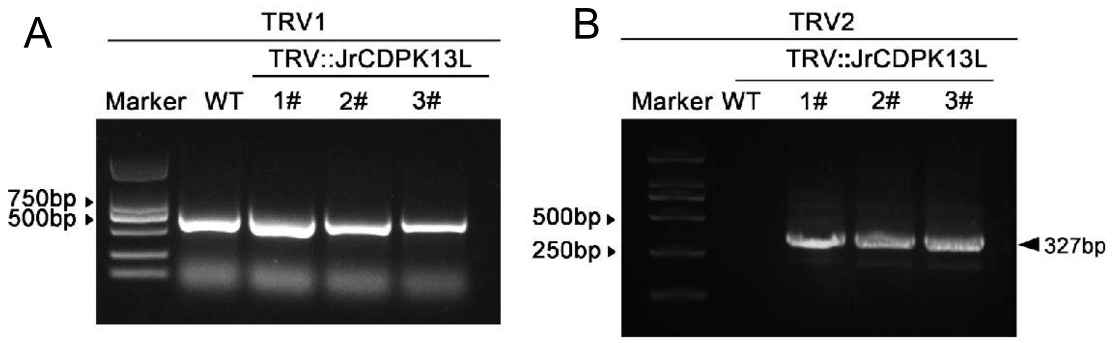

Supplementary Figure S3. The PCR identification of RNA1 and RNA2 of TRV::CDPK13L in walnut leaves. pTRV1-F/R and pTRV2-F/R were used for PCR validation in WT samples, and pTRV1-F/R and pTRV2-F/JrCDPK13L-R were used for PCR validation in *TRV::JrCDPK13L* samples.

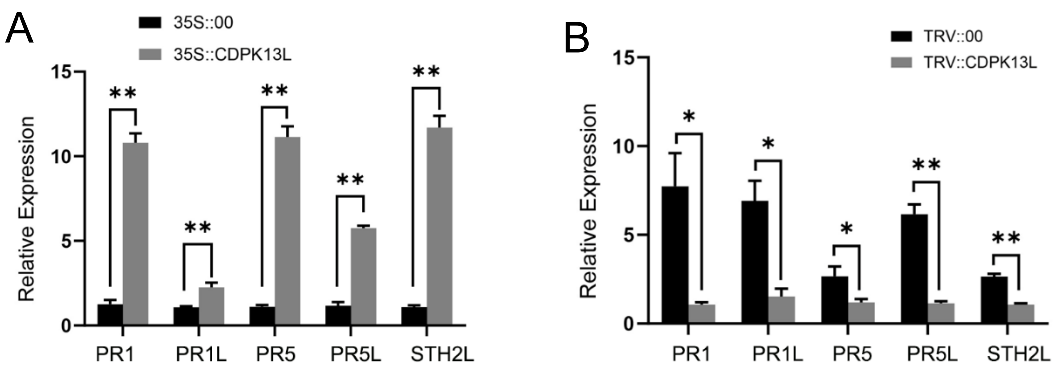

Supplementary Figure S4. The relative expression levels of PR genes (*JrPR1*, *JrPR1L*, *JrPR5*, *JrPR5L*, and *JrSTH2L*) in both overexpression (35S::JrCDPK13L and 35S::00) and silencing (TRV::JrCDPK13L and TRV::00) backgrounds. 18S rRNA was amplified as an internal control. Data are presented as means ± SD of three biological replicates. Asterisks indicate significant differences compared with the WT (two-tailed Student's t-test, *P<0.05, **P<0.01; ns, no significance, P>0.05).

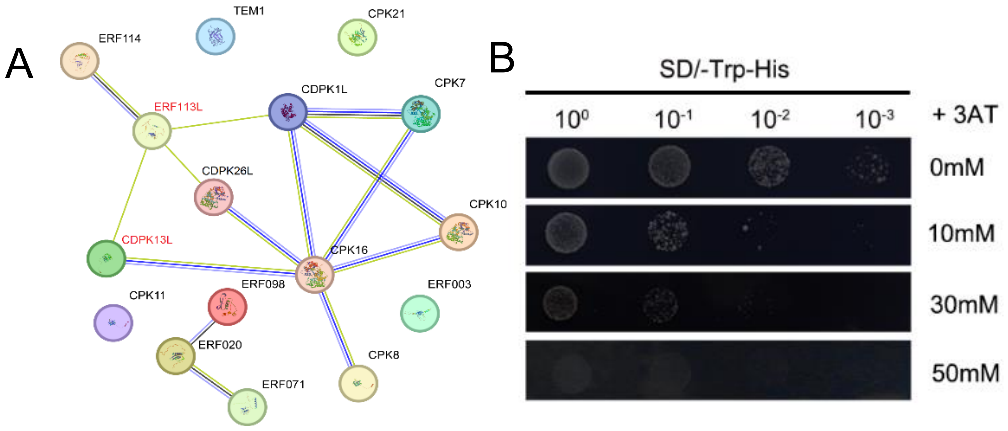

Supplementary Figure S5. Screening and suppression of auto-activation activity of JrERF113L. (A) Analysis of protein-protein interaction network between CDPK protein and ERF transcription factors. (B) Yeast strains transformed with pGBKT7-JrERF113L were cultured on SD/-Trp-His medium supplemented with different concentrations of 3-AT. It was determined that 50 mM 3-AT completely suppressed its auto-activation activity.

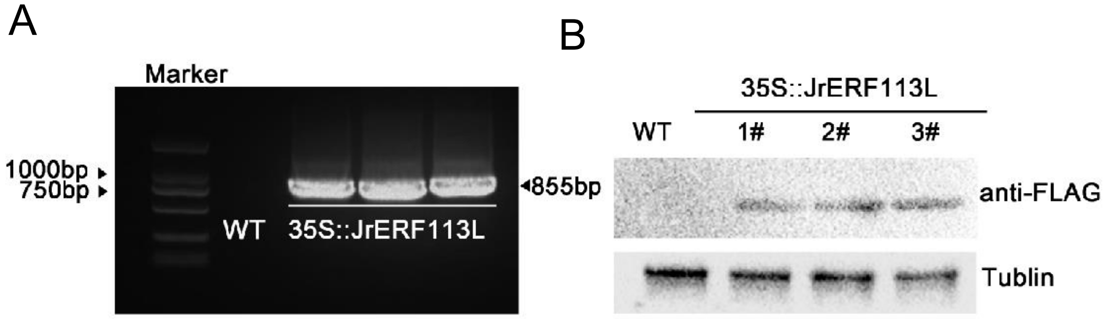

Supplementary Figure S6. The presence of the transgene in *35S::JrERF113L* walnut leaves was detected by PCR amplification (A) and western-blot with FLAG antibody (B).

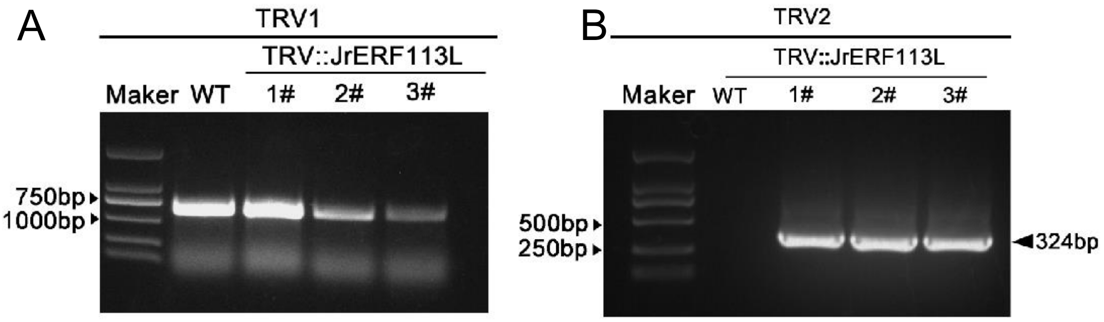

Supplementary Figure S7. The PCR identification of RNA1 and RNA2 of *TRV::CDPK13L* in walnut leaves. pTRV1-F/R and pTRV2-F/R were used for PCR validation in WT samples, and pTRV1-F/R and pTRV2-F/JrERF113L-R were used for PCR validation in *TRV::JrERF113L* samples.

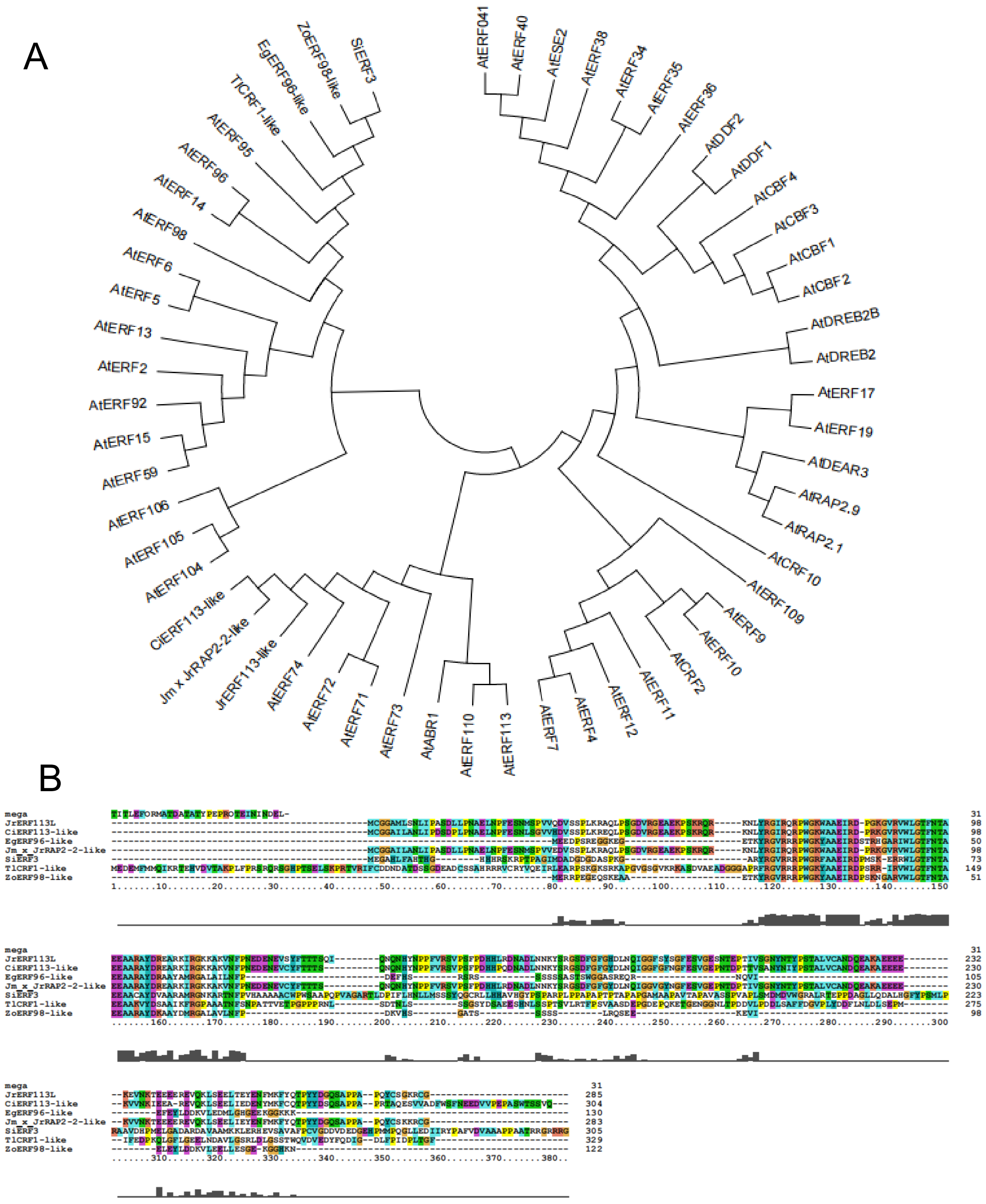

Supplementary Figure S8. Bioinformatic Characterization of JrERF113L. (A) Phylogenetic Analysis of JrERF113L and Domain Comparison (B) with Homologous Proteins.

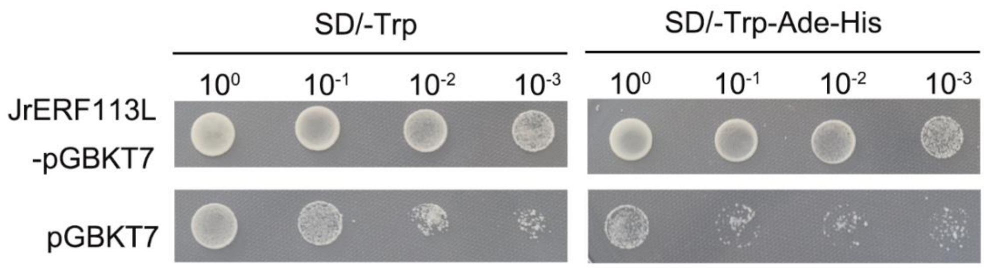

Supplementary Figure S9. Transcriptional auto-activation of JrERF113L. The growth of yeast transformed with JrERF113L-pGBKT7 on SD/-Trp-His-Ade medium demonstrates its transcriptional auto-activation activity.

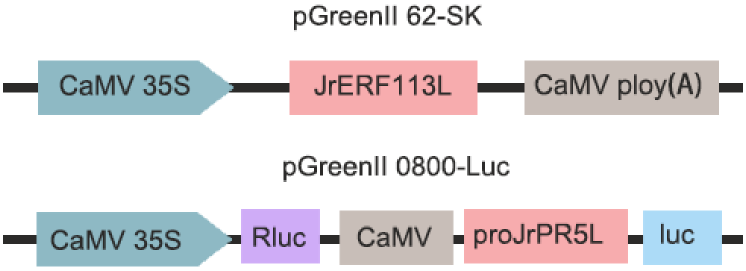

Supplementary Figure S10. Schematic diagram of the vector constructs for the dual-luciferase reporter assay (JrERF113L-pGreenII62-SK, *proJrPR5L*-pGreenII 0800-LUC). (Top) Effector construct: The coding sequence of JrERF113L was cloned into the pGreenII62-SK vector, driven by the 35S promoter. (Bottom) Reporter construct: The promoter sequence of *JrPR5L* was cloned into the pGreenII 0800-LUC vector to drive the expression of the firefly luciferase reporter gene.

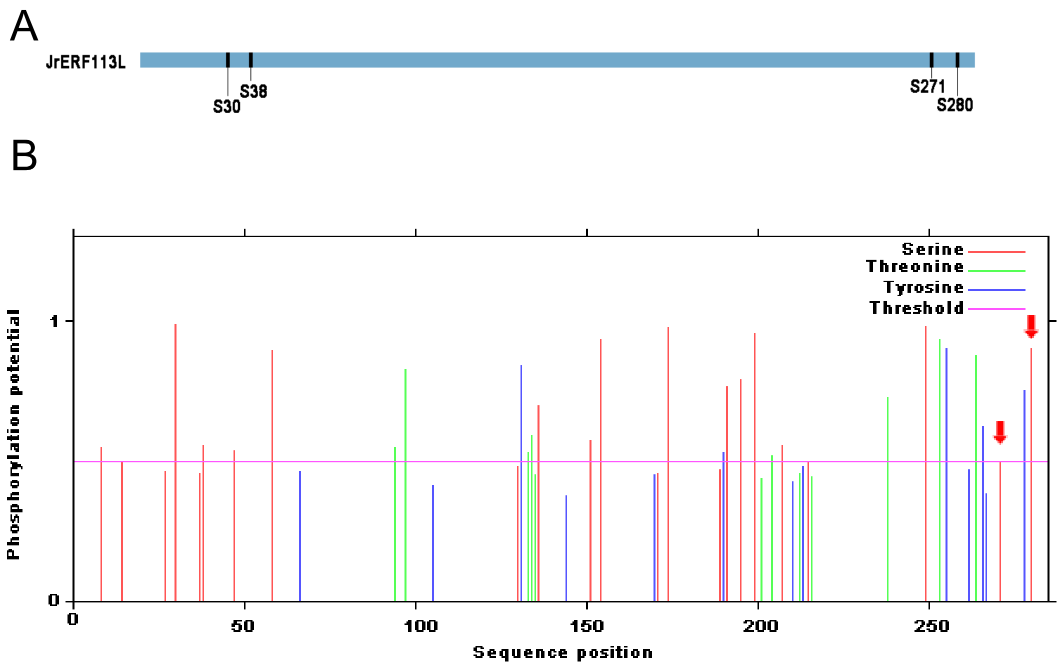

Supplementary Figure S11. Predicted phosphorylation sites in the JrERF113L protein. Putative phosphorylation sites in the JrERF113L protein sequence were predicted using the relevant kinase group models (AGC, CaMK) in the GPS 6.0 database and the netphos 3.1 server. Red arrows indicate two serine residues (Ser271 and Ser280) identified as high-confidence predictions after screening.

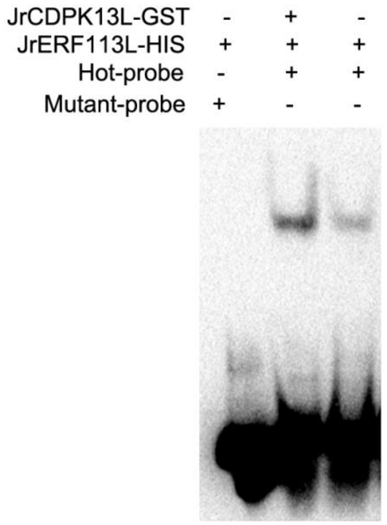

Supplementary Figure S12. Phosphorylation of JrERF113L by JrCDPK13L enhances its binding affinity to the *JrPR5L* promoter. EMSA showed a marked increase in JrERF113L binding to the *JrPR5L* promoter following pre-incubation with JrCDPK13L (Lane 2) relative to the non-phosphorylated control (Lane 3).

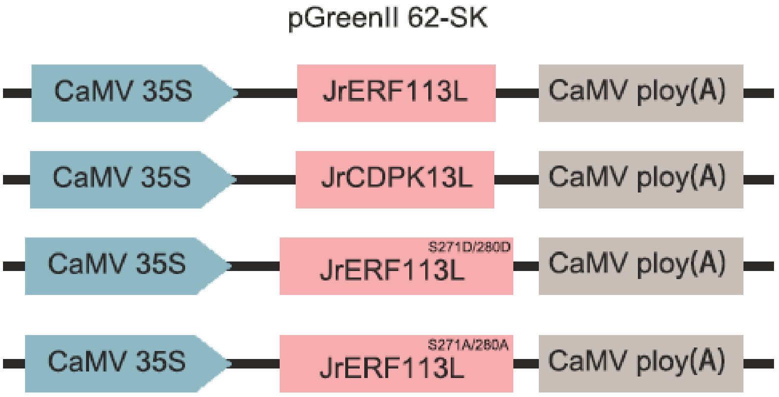

Supplementary Figure S13. Schematic representation of the vector constructs for the dual-luciferase assay (JrERF113L, JrCDPK13L, JrERF113L$^{271D/280D}$, JrERF113L$^{271A/280A}$-pGreenII 62-SK).

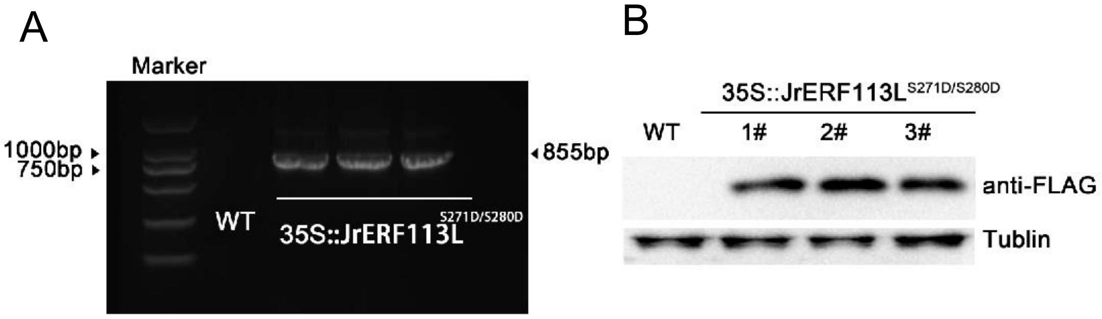

Supplementary Figure S14. The presence of the transgene in *35S:JrERF113L* $^{S271D/S280D}$ walnut leaves was detected by PCR amplification (A) and western-blot with FLAG antibody (B).

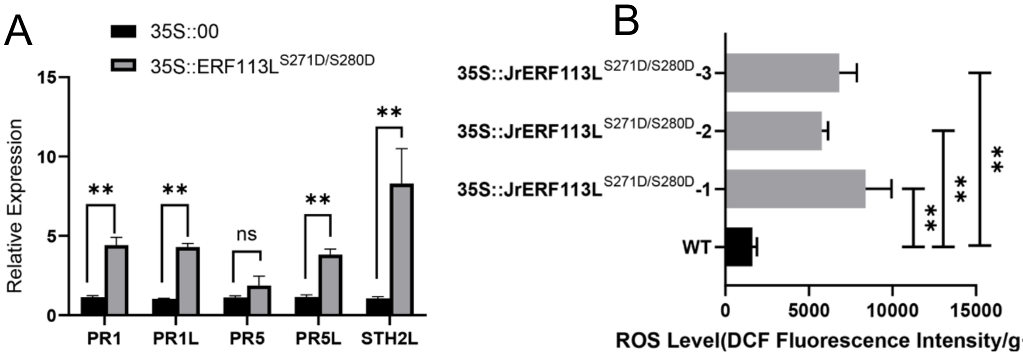

Supplementary Figure S15. Activation of defense responses in *35S:JrERF113L* $^{S271D/S280D}$ leaves. (A) qRT-PCR analysis of the relative expression levels of PR genes in *35S::JrERF113L*$^{S271D/S280D}$ VS. *35S::00.* (B) The reactive oxygen species (ROS)

production in JrERF113L$^{S271D/S280D}$ walnut leaves. 18S rRNA was amplified as an internal control. Data are presented as means ± SD of three biological replicates. Asterisks indicate significant differences compared with the control (two-tailed Student's t-test, *P < 0.05, P < 0.01; ns, no significance, P>0.05).

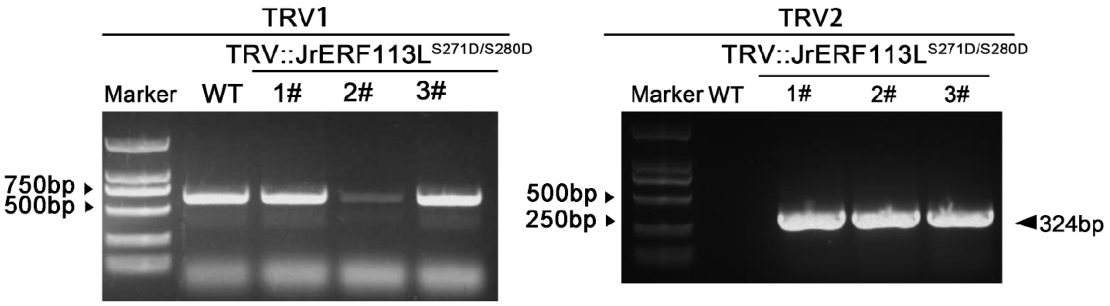

Supplementary Figure S16. The PCR identification of RNA1 and RNA2 of *TRV::JrERF113L*$^{S271D/S280D}$ in walnut leaves. pTRV1-F/R and pTRV2-F/R were used for PCR validation in WT samples, and pTRV1-F/R and pTRV2-F/JrERF113L-R were used for PCR validation in *TRV::JrERF113L*$^{S271D/S280D}$ samples.

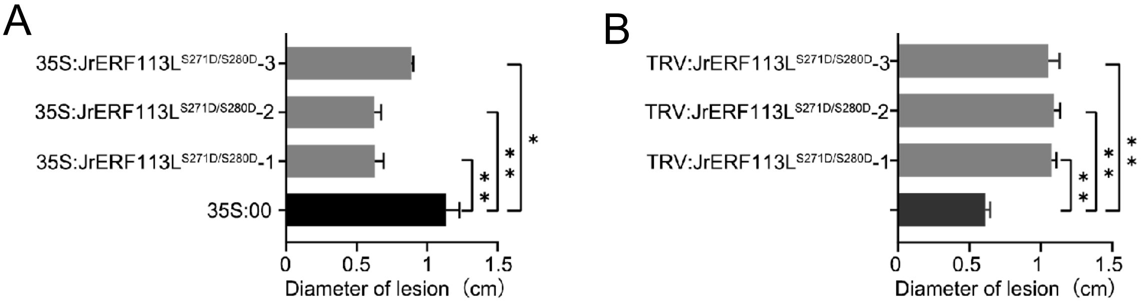

Supplementary Figure S17. Diameter of lesions in 35S::JrERF113L$^{S271D /S280D}$ (A) and TRV::JrERF113L$^{S271D/S280D}$ (B) walnut leaves. Asterisks indicate significant differences compared with the WT (two-tailed Student's t-test, *P<0.05, **P<0.01).
